# US healthcare professionals’ knowledge, attitudes, and practices regarding RSV disease and vaccination in adults during the 2024–2025 RSV season

**DOI:** 10.1371/journal.pone.0353266

**Published:** 2026-07-22

**Authors:** Elizabeth M. La, Kyli Gallington, David Singer, Tsion Fikre, Noha Eltoukhy, Yipin Han, Zaneta Balantac, Donald M. Bushnell

**Affiliations:** 1 US Health Economics and Outcomes Research, GSK, Philadelphia, Pennsylvania, United States of America; 2 PPD^TM^ Evidera^TM^ Patient Centered Research, Thermo Fisher Scientific, Waltham, Massachusetts, United States of America; 3 US Medical Affairs, GSK, Philadelphia, Pennsylvania, United States of America; University of North Dakota School of Medicine and Health Sciences, UNITED STATES OF AMERICA

## Abstract

**Background:**

Adults aged ≥60 years and adults with certain chronic medical conditions are at increased risk for severe respiratory syncytial virus (RSV) disease. However, RSV vaccine uptake in the United States (US) remains low, with limited awareness of RSV among adults and potential knowledge gaps among healthcare professionals (HCPs). This study assessed US HCP knowledge, attitudes, and practices (KAP) related to RSV disease and vaccination.

**Methods:**

A cross-sectional, web-based survey was administered among 700 HCPs, including primary care physicians (PCPs), specialist physicians, nurse practitioners (NPs) and physician assistants (PAs), and pharmacists, between December 2024–January 2025. Survey questions assessed RSV disease- and vaccination-related KAP, with descriptive statistics calculated overall and by HCP type. Characteristics associated with RSV-related KAP were identified through multivariable logistic regression modeling.

**Results:**

Among the 700 respondents (199 PCPs, 153 specialist physicians, 148 NPs and PAs, and 200 pharmacists), 72.3% reported being very familiar with RSV disease. However, potential knowledge gaps were identified (e.g., related to approved indications for RSV vaccines and Advisory Committee on Immunization Practices [ACIP] recommendations). HCPs commonly cited patients’ refusal or hesitation (62.3%) and patients’ lack of awareness of RSV (58.0%) as barriers to recommending and administering RSV vaccines. Although only 36.4% of HCPs reported that their workplace stocked RSV vaccine(s), 84.1% reported recommending RSV vaccines to eligible adult patients. However, RSV vaccination recommendations were not consistently implemented among all eligible adult patients. In multivariable modeling, characteristics that were significantly associated with RSV vaccination practices included familiarity with RSV disease, knowledge of ACIP recommendations, RSV vaccine stocking, and use of clinical decision supports, among other characteristics.

**Conclusions:**

Potential knowledge- and practice-related gaps remain regarding adult RSV disease and vaccination among US HCPs. Continued efforts are needed to enhance RSV-related education among HCPs and ensure eligible patients are able to access RSV vaccination.

## Introduction

Respiratory syncytial virus (RSV) is a common cause of acute respiratory illness worldwide and across age groups [1]. While RSV infection in adults most frequently results in mild, cold-like symptoms, adults aged ≥60 years and adults with certain chronic medical conditions are at increased risk for severe RSV disease [1,2]. In the United States (US), a recent modeling analysis estimated that RSV contributes to approximately 178,000 hospitalizations and 14,000 deaths annually among adults aged ≥60 years [3].

Three RSV vaccines were approved by the US Food and Drug Administration (FDA) between 2023–2024 (AREXVY^®^ [GSK], ABRYSVO^®^ [Pfizer, Inc], and MRESVIA^®^ [Moderna, Inc]) for adults aged ≥60 years, and subsequently for younger adults who are at increased risk for severe RSV disease [4–7]. RSV vaccination recommendations from the US Centers for Disease Control and Prevention’s (CDC’s) Advisory Committee on Immunization Practices (ACIP) have changed over time since vaccine introduction. In June 2023, the ACIP recommended RSV vaccination for adults aged ≥60 years under shared clinical decision-making [8]. The following year, in June 2024, the ACIP revised their recommendation to include all adults aged ≥75 years and adults aged 60–74 years at increased risk for severe RSV disease [9]. Most recently, the ACIP updated their risk-based recommendation to also include those aged 50–59 years, which took effect in June 2025 [10].

Previous research has highlighted potential knowledge and practice gaps related to RSV disease and vaccination among healthcare professionals (HCPs). In a November 2023 survey, one-third of US HCPs reported not recommending, prescribing, or administering RSV vaccines to any patient aged ≥60 years in the past three months; one-quarter were unaware of FDA-approved indications and ACIP recommendations for RSV vaccination among older adults; and nearly half worried that they were not initiating conversations about RSV vaccination with all of their increased-risk older adult patients [11]. Results from a global systematic literature review spanning 2019–2024 suggested a somewhat limited awareness of RSV disease and its prevention among HCPs, though HCPs favorably viewed vaccination as a preventative measure [12]; similar findings were observed across studies conducted in the US and internationally, suggesting that RSV-related knowledge among HCPs may represent a global area for improvement [12]. In Germany and Italy, for example, a 2023 study found that nearly three-quarters of surveyed HCPs indicated they needed more information regarding RSV, compared with <35% for other included respiratory infections [13].

Despite the availability of RSV vaccines since 2023, vaccination uptake among US adults has remained relatively low. According to a recent analysis of claims data from August 2023–February 2025, only 16.4% of all adults aged ≥60 years had received an RSV vaccine [14]. In comparison, among adults aged ≥65 years, the CDC estimated influenza vaccination uptake at 63.8% during the 2024–2025 influenza season and pneumococcal vaccination uptake at 70.3% as of 2024 [15,16]. Recent changes in the RSV vaccination landscape and the suboptimal uptake of RSV vaccines among US adults highlight a need to reassess HCPs’ knowledge, attitudes, and practices (KAP) related to adult RSV disease and vaccination.

This study aimed to assess RSV-related KAP and identify key characteristics associated with RSV-related KAP among US HCPs, including primary care physicians (PCPs), specialist physicians, nurse practitioners (NPs) and physician assistants (PAs), and pharmacists. Findings from this study can provide valuable insights into potential gaps and challenges associated with RSV vaccine implementation in real-world practice, which can help to inform targeted interventions and education for HCPs and their patients.

**Fig 1:**
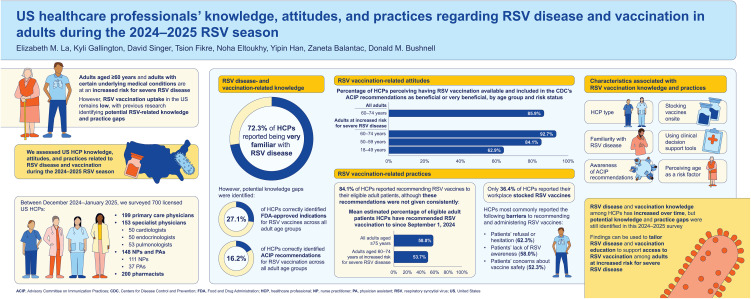
Graphical summary of the study results.

A graphical summary of the study results is provided in Fig 1.

## Methods

### Study design and population

This cross-sectional study used a web-based, self-administered survey targeting 700 US HCPs to evaluate their RSV disease- and vaccination-related KAP. Participants were recruited using MedPanel, Inc.’s network of practicing HCPs [17].

A stratified sampling approach was used to identify the population of interest, with targets set by HCP type: 200 PCPs, 150 specialist physicians (50 cardiologists, 50 endocrinologists, and 50 pulmonologists), 150 NPs and PAs, and 200 pharmacists. Sampling targets were used to ensure sufficient subgroup sizes for meaningful analyses.

Participants were invited via email and directed to the survey, where they first consented to an eligibility screening; if eligible, participants were asked to provide their full informed consent. The survey was expected to take approximately 20 minutes, and respondents could stop at any point and resume at a later time. Survey responses were collected between December 30, 2024–January 31, 2025 (until 700 surveys were completed). HCPs received honoraria for their time spent completing the survey.

#### Survey eligibility.

Eligible US participants were licensed HCPs in one of the included categories. HCPs, excluding pharmacists, had to have provided care for ≥1 patient aged ≥50 years in the past week and work ≥21 hours per week in direct patient care (clinical setting, including outpatient). Pharmacists had to have personally interacted with ≥1 patient aged ≥50 years in the past week and work ≥21 hours per week in a pharmacy, as well as be certified with the legal authority to administer immunizations and work in a large pharmacy chain, grocery store/chain, mass merchant, regional pharmacy chain, or independent pharmacy. All HCPs had to have been working in their current profession for ≥2 years, have the ability to complete the survey in English, and provide informed consent.

HCPs were excluded if they had resident, fellow, or trainee status, or worked in a state prohibiting monetary compensation for participation. Pharmacists were excluded if they reported working for a mail-order, hospital-based, specialty, or “other” pharmacy setting. Other HCPs were excluded if they selected their primary work environment as a hospital-based inpatient or “other” setting (e.g., research, administration) or spent most of their time in an emergency care or surgical setting. Participants who were straight-liners (selected the same answer consistently for most of the survey) or speeders (completed the survey in <7 minutes) were excluded from the final sample.

### Variables

The primary objectives of the survey were to describe HCPs’ knowledge of adult RSV disease, including risk factors for severe RSV disease; knowledge of adult RSV vaccination, including approved indications and recommendations, as well as vaccine efficacy; attitudes regarding adult RSV disease and vaccination, including importance of prevention; and practices regarding adult RSV vaccination, including engaging in vaccination discussions and providing vaccination recommendations. As exploratory objectives, the survey also included questions to explore whether differences existed between HCPs in their RSV-related KAP, as well as to describe other vaccination-related practices. The full survey, including screener questions, is available in S1 File.

### Statistical analysis

Analyses were conducted using SAS 9.4 statistical software (SAS, Cary, NC) following receipt of final data on February 18, 2025. Missing data were not imputed for analyses. Descriptive summary statistics were presented among the overall cohort and stratified by HCP type for the primary analysis. Categorical variables were presented as the number of available observations (n) and the percentage for each category, where the denominator (N) was the number of participants who answered the question. For continuous variables, results were presented as mean (standard deviation [SD]).

Multivariable logistic regression models were used to further explore HCP characteristics associated with key outcomes of interest. Univariate logistic regressions were first performed to examine potential covariates for model inclusion. Covariates with *P* ≤ 0.05 were then included in the multivariable models. Odds ratios (ORs) and corresponding 95% confidence intervals (CIs) were reported.

The analytic sample, using analysis of variance (ANOVA), fixed effects with four strata (i.e., HCP type), effect size of 0.20, alpha of 0.05, and power of 0.95 required 436 completed surveys. For the exploratory analysis, a 1-tailed test with an OR ≥1.3, alpha of 0.05, and power of 0.90 required 604 completed surveys. Data collection remained open until 700 surveys were completed.

### Ethics

This study complied with all applicable laws regarding subject privacy. No direct subject contact or primary collection of individual subject data occurred. Study results are in tabular form and presented as aggregate analyses that omit subject identification. Eligible respondents were required to read an informed consent form and provide written electronic acknowledgment of consent before proceeding to survey questions. The Salus Institutional Review Board (IRB) determined that this study met the criteria for exemption from IRB review in accordance with 45 CFR 46.104(d)(2(i)) on November 23, 2024.

## Results

### Participant characteristics

A total of 26,482 invitations were sent; of the 1,857 respondents who began the survey, 1,463 completed the screening and 778 were eligible for the survey. Of these, six participants did not provide consent, 58 did not complete the survey, and 14 were considered straight-liners or speeders and were excluded. The final sample included 700 respondents: 199 PCPs, 153 specialist physicians (n = 50 cardiologists, n = 50 endocrinologists, and n = 53 pulmonologists), 148 NPs and PAs (n = 111 and n = 37, respectively), and 200 pharmacists (S1 Fig).

Overall, two-thirds of HCPs (66.6%) identified as White and approximately half (51.3%) as female. Mean (SD) HCP age was 49.6 (12.0) years, with 73.1% reporting ≥11 years of experience in their primary medical profession. Most HCPs reported working in a suburban (49.6%) or urban (36.6%) area (Tables 1 and S1).

**Table 1:** Summary of demographic characteristics, overall and by HCP type.

Characteristic, n (%)	Overall (N = 700)	PCPs (N = 199)	Specialists (N = 153)	NPs and PAs (N = 148)	Pharmacists (N = 200)
Primary medical profession					
PCP	199 (28.4)	199 (100.0)	N/A	N/A	N/A
Cardiologist	50 (7.1)	N/A	50 (32.7)	N/A	N/A
Endocrinologist	50 (7.1)	N/A	50 (32.7)	N/A	N/A
Pulmonologist	53 (7.6)	N/A	53 (34.6)	N/A	N/A
NP	111 (15.9)	N/A	N/A	111 (75.0)	N/A
PA	37 (5.3)	N/A	N/A	37 (25.0)	N/A
Pharmacist	200 (28.6)	N/A	N/A	N/A	200 (100.0)
Gender identity					
Male	333 (47.6)	119 (59.8)	111 (72.5)	18 (12.2)	85 (42.5)
Female	359 (51.3)	77 (38.7)	40 (26.1)	128 (86.5)	114 (57.0)
Gender identity not listed	8 (1.1)	3 (1.5)	2 (1.3)	2 (1.4)	1 (0.5)
Age, mean (SD)					
	N = 699	N = 198	N = 153	N = 148	N = 200
	49.6 (12.0)	53.2 (11.9)	53.4 (12.1)	47.6 (11.2)	44.5 (10.4)
Years in practice					
2–10 years	188 (26.9)	41 (20.6)	35 (22.9)	66 (44.6)	46 (23.0)
11–20 years	213 (30.4)	51 (25.6)	40 (26.1)	45 (30.4)	77 (38.5)
21–30 years	196 (28.0)	66 (33.2)	53 (34.6)	30 (20.3)	47 (23.5)
> 30 years	103 (14.7)	41 (20.6)	25 (16.3)	7 (4.7)	30 (15.0)
Race and ethnicity^a^					
African American or Black	27 (3.9)	7 (3.5)	0 (0.0)	10 (6.8)	10 (5.0)
American Indian or Alaska Native	2 (0.3)	0 (0.0)	0 (0.0)	0 (0.0)	2 (1.0)
Asian	171 (24.4)	62 (31.2)	42 (27.5)	13 (8.8)	54 (27.0)
Hispanic or Latino	31 (4.4)	14 (7.0)	7 (4.6)	6 (4.1)	4 (2.0)
Middle Eastern or North African	9 (1.3)	2 (1.0)	4 (2.6)	0 (0.0)	3 (1.5)
Native Hawaiian or Pacific Islander	3 (0.4)	0 (0.0)	1 (0.7)	0 (0.0)	2 (1.0)
White	466 (66.6)	115 (57.8)	100 (65.4)	120 (81.1)	131 (65.5)
Other	24 (3.4)	8 (4.0)	8 (5.2)	2 (1.4)	6 (3.0)
US Census region of primary work^b^					
Midwest	144 (20.6)	39 (19.6)	33 (21.6)	23 (15.5)	49 (24.5)
Northeast	134 (19.1)	38 (19.1)	32 (20.9)	26 (17.6)	38 (19.0)
South	237 (33.9)	63 (31.7)	51 (33.3)	55 (37.2)	68 (34.0)
West	185 (26.4)	59 (29.6)	37 (24.2)	44 (29.7)	45 (22.5)
Location of HCP workplace					
Urban (i.e., city or metropolitan area)	256 (36.6)	64 (32.2)	74 (48.4)	48 (32.4)	70 (35.0)
Suburban (i.e., large residential area that surrounds a main city)	347 (49.6)	108 (54.3)	69 (45.1)	73 (49.3)	97 (48.5)
Rural	97 (13.9)	27 (13.6)	10 (6.5)	27 (18.2)	33 (16.5)

Note: Additional HCP sample characteristics are available in S1 Table.

^a^Not mutually exclusive.

^b^HCPs currently residing in Maine, Massachusetts, Minnesota, or Vermont were not eligible to complete the survey as these states do not allow HCPs to be compensated for participation.

Abbreviations: HCP, healthcare professional; N/A, not applicable; NP, nurse practitioner; PA, physician assistant; PCP, primary care physician; SD, standard deviation; US, United States.

### HCP knowledge of adult RSV disease

**Fig 2:**
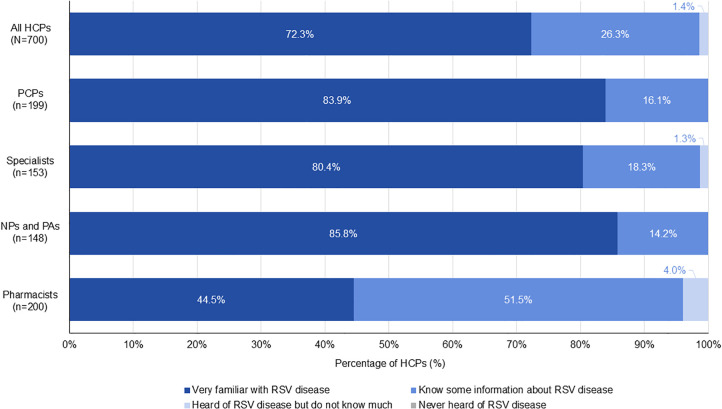
HCPs’ familiarity with RSV disease, by HCP type. Abbreviations: HCP, healthcare professional; NP, nurse practitioner; PA, physician assistant; PCP, primary care physician; RSV, respiratory syncytial virus.

In the overall sample (N = 700), HCPs most frequently reported being very familiar with RSV disease (72.3%) or knowing some information about RSV disease (26.3%). Familiarity with RSV disease differed significantly by HCP type, with NPs and PAs most frequently reporting being very familiar with RSV disease (85.8%), followed by PCPs (83.9%), specialist physicians (80.4%), and pharmacists (44.5%; Fig 2).

When asked about risk factors for severe RSV disease, HCPs generally perceived increasing age to be associated with a higher risk for severe RSV disease. Specifically, 59.4% reported that adults aged 50–59 years were at moderate risk for severe RSV disease, while the majority of HCPs reported that adults aged 60–74 years (60.3%) and adults aged ≥75 years (87.3%) were at high risk for severe RSV disease (S2a Fig).

HCPs were also surveyed on the extent to which certain medical conditions are risk factors for severe RSV disease. Risk factors that were most frequently selected by HCPs as being “high risk” for severe RSV disease among adult patients included moderate or severe immunocompromise (88.0%), chronic lung or respiratory disease (87.3%), and neurological or neuromuscular conditions causing impaired airway clearance or respiratory muscle weakness (69.0%). In contrast, the least frequently selected risk factors were severe obesity (38.4%), chronic liver disease (37.9%), and chronic hematologic conditions (33.3%; S2b Fig).

### HCP knowledge of adult RSV vaccination

Approximately one-quarter of HCPs (25.1%) correctly identified that there were three FDA-approved RSV vaccines for use in adults at the time of the survey. When asked which non-pregnant adult groups were included in the FDA-approved indications for RSV vaccines, less than half of HCPs (45.0%) correctly reported that there was at least one RSV vaccine approved for adults aged 18–49 years at increased risk for severe RSV disease, and approximately half (52.6%) correctly reported the same for adults aged 50–59 years at increased risk for severe RSV disease. Over one-third of HCPs (39.4%) were incorrect or did not know about the FDA-approved indications for RSV vaccines among all adults aged 60–74 years; 14.6% were incorrect or unsure regarding those aged ≥75 years. Overall, 27.1% of HCPs correctly identified all FDA-approved indications for RSV vaccines among adults across all age groups.

Among the 698 HCPs who were aware that FDA-approved indications existed for adult RSV vaccines, 80.5% correctly identified the ACIP recommendation for all adults aged ≥75 years. Substantially fewer HCPs correctly identified the risk-based recommendation for adults aged 60–74 years (34.8%), and only 21.2% and 32.1% correctly identified that RSV vaccines were not ACIP-recommended at the time of the survey for any adults aged 50–59 years and 18–49 years, respectively. In fact, approximately half of HCPs incorrectly answered that RSV vaccines were ACIP-recommended among those aged 50–59 years (59.0%) and 18–49 years (44.3%). Among those aware of an FDA-approved indication for adult RSV vaccines, 16.2% correctly identified all ACIP recommendations.

HCPs were also asked to estimate the vaccine efficacy of the available RSV vaccines among adults aged ≥60 years during the first season after vaccination. Overall, 22.3% of HCPs correctly identified RSV vaccine efficacy as ≥75%; most HCPs underestimated (55.9%) or did not know (21.9%) the efficacy of available RSV vaccines (S2 Table).

Additional results related to HCPs’ RSV disease and vaccine knowledge are available in S2 Table.

### HCP attitudes toward adult RSV disease and vaccination

**Fig 3:**
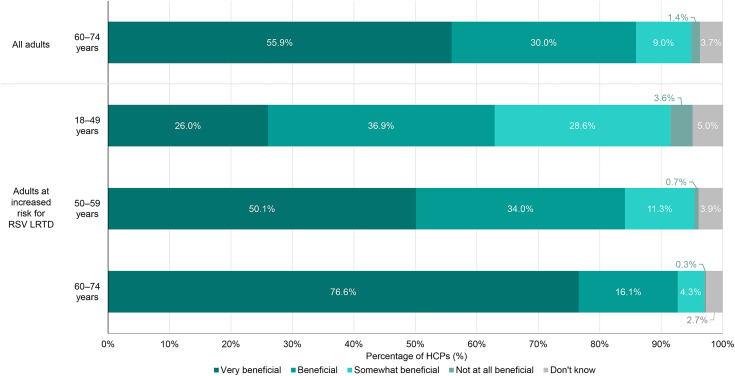
HCPs’ perceived benefit of having RSV vaccination available and included in the CDC’s ACIP recommendations. Abbreviations: ACIP, Advisory Committee on Immunization Practices; CDC, Centers for Disease Control and Prevention; HCP, healthcare professional; LRTD, lower respiratory tract disease; RSV, respiratory syncytial virus.

HCPs’ perceived benefit of having RSV vaccines available and included in ACIP recommendations was assessed by vaccine recipient age and risk status. Specifically, 85.9% of HCPs viewed RSV vaccination as beneficial or very beneficial for all adults aged 60–74 years. For increased-risk adults aged 50–59 years and 18–49 years, RSV vaccination was viewed as beneficial or very beneficial by 84.1% and 62.9% of HCPs, respectively (Fig 3).

Additional results related to HCPs’ RSV vaccination attitudes are available in S3 Table.

### HCP practices related to adult RSV vaccination

#### Recommending vaccines.

**Fig 4:**
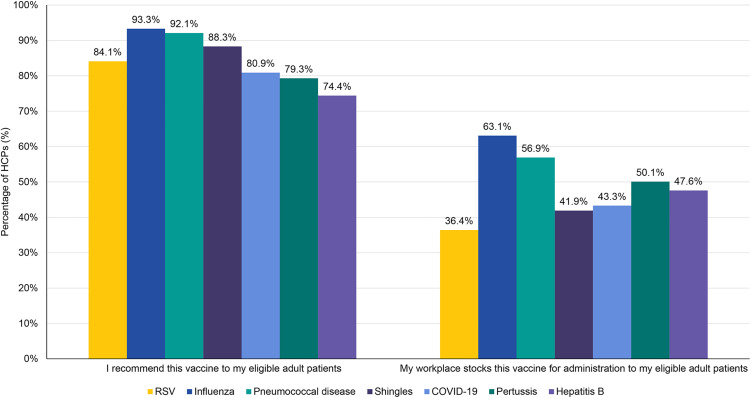
Vaccination recommendation and stocking practices among HCPs. Note: Stratification by HCP type is available in S5 Table. Abbreviations: COVID-19, coronavirus disease 2019; HCP, healthcare professional; RSV, respiratory syncytial virus.

Regarding real-world practices, a lower percentage of HCPs reported recommending RSV vaccines to their eligible adult patients (84.1%) compared with influenza (93.3%), pneumococcal (92.1%), and shingles (88.3%) vaccines (Fig 4).

The percentage of patients to whom HCPs reported discussing, recommending, and/or administering RSV vaccines since September 1, 2024 varied across age groups. HCPs reported recommending RSV vaccination to a mean (SD) of 53.7% (36.5%) of adults aged 60–74 years at increased risk for severe RSV disease and 58.8% (37.1%) of all adults aged ≥75 years. On average, across all age groups, the proportion of patients with whom HCPs reported discussing and recommending RSV vaccination was highest among PCPs and lowest among pharmacists, while vaccine administration was highest among pharmacists. Moreover, HCPs estimated that the mean (SD) follow-through vaccination rate among patients to whom they recommended RSV vaccination was 48.9% (26.2%) for adults aged 60–74 years and 59.3% (27.1%) for adults aged ≥75 years.

When asked about approaches or processes used to inform decision-making for RSV vaccination recommendations among adults aged ≥60 years, HCPs most frequently reported using shared clinical decision-making (68.4%), clinical judgment (68.3%), prompts from their patients (61.0%), and ACIP recommendations (60.1%; S4 Table).

Overall, 34.0% of HCPs (including 53.0% of pharmacists) noted their workplace requires an ACIP recommendation to be able to recommend and/or administer FDA-approved adult vaccines (S5 Table).

#### Stocking vaccines.

Only 36.4% of HCPs reported that their workplace stocked RSV vaccines, the lowest percentage among all included vaccine-preventable diseases (Fig 4). However, 80.3% agreed or strongly agreed that stocking RSV vaccines would help ensure at-risk individuals have access to vaccination. Stocking practices significantly varied by HCP type, with 70.5% of pharmacists reporting their workplaces stocked RSV vaccines, compared with 29.6% of PCPs, 18.9% of NPs and PAs, and 17.6% of specialist physicians (S5 Table).

#### Barriers or concerns related to RSV vaccination.

**Fig 5:**
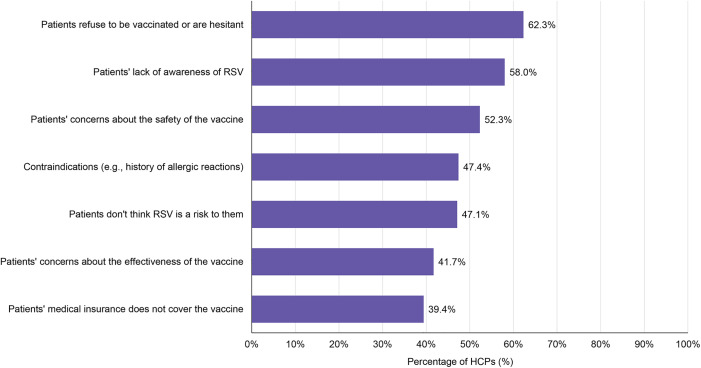
Barriers or concerns related to recommending and administering RSV vaccination among HCPs. Note: Other selected barriers included: “I have concerns about vaccine effectiveness” (10.3%), “I have concerns about vaccine safety” (9.0%), “I do not believe RSV is as severe as other conditions and would prioritize other vaccines” (7.1%), “My workplace does not focus on recommending or administering vaccinations” (6.3%), “I do not regularly review patients’ vaccination records as part of their visit” (4.7%), and “None of the above” (2.1%). Abbreviations: HCP, healthcare professional; RSV, respiratory syncytial virus.

Overall, when asked about barriers or concerns related to recommending or administering RSV vaccines, HCPs most frequently cited patients’ refusal or hesitation (62.3%), patients’ lack of awareness of RSV (58.0%), and patients’ concerns about the safety of the vaccine (52.3%; Fig 5).

### Characteristics associated with adult RSV vaccination knowledge and practices

Using multivariable adjusted models, the likelihood (OR [95% CI]) of HCPs not being aware of the current RSV vaccine ACIP recommendations among adults aged ≥60 years was assessed. The adjusted odds of not being fully knowledgeable about ACIP recommendations were 1.82-fold (1.03–3.19) higher among NPs and PAs, and 0.48-fold (0.29–0.79) lower among pharmacists, compared with the reference group of PCPs (i.e., compared with PCPs, pharmacists were more likely to be knowledgeable while NPs and PAs were less likely). Additionally, HCPs whose workplaces did not stock RSV vaccines were less likely to be fully knowledgeable of relevant ACIP recommendations (0.66 [0.45–0.97]) than HCPs whose workplaces did stock them.

Adjusted analyses were also performed to examine characteristics associated with the likelihood of HCPs being in the bottom quartile for the percentage of eligible patients recommended RSV vaccination. HCPs who reported recommending RSV vaccination to ≤15% of their patients aged 60–74 years at increased risk for severe RSV disease and ≤20% of all their patients aged ≥75 years fell within the bottom quartile. The adjusted odds of being in this bottom quartile were higher among HCPs who were less familiar with RSV disease (2.87 [1.90–4.33]), were not fully knowledgeable about ACIP recommendations (3.17 [1.86–5.41]), and did not report use of clinical decision support systems or patient questionnaires on vaccine history to help inform recommendations (3.51 [1.19–10.30]). In contrast, the odds of being in the bottom quartile were lower if HCPs reported that RSV vaccines were stocked at their workplace (0.61 [0.39–0.94]) and if HCPs perceived patient age (≥60 years) as a moderate-to-high risk factor for severe RSV disease (0.29 [0.14–0.63]).

**Fig 6:**
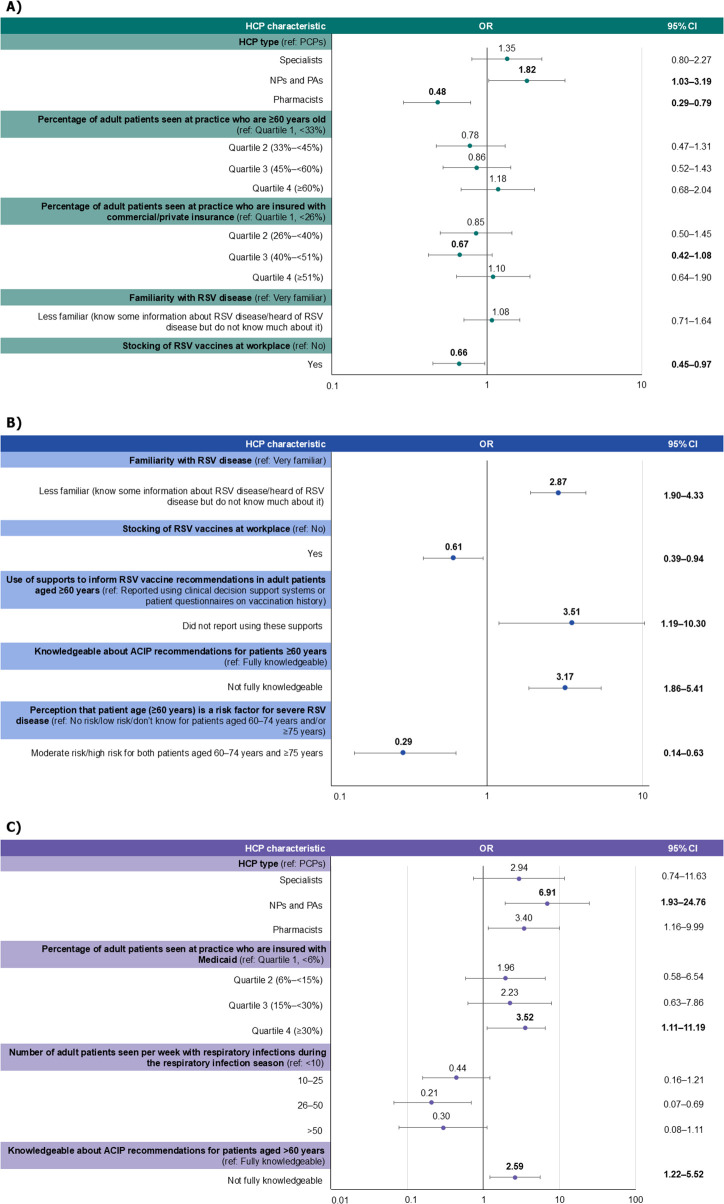
Characteristics associated with RSV vaccination knowledge and practices among HCPs. **A)** Likelihood of not being fully knowledgeable about RSV vaccine ACIP recommendations for patients aged ≥60 years (N = 698);^a^ **B)** Likelihood of being in the bottom quartile for percentage of patients recommended RSV vaccination since September 1, 2024 in both patients aged 60–74 years who are at increased risk for severe RSV disease and all patients aged ≥75 years (N = 700);^b^ **C)** Likelihood of being in the bottom quartile for percentage of patients administered RSV vaccination since September 1, 2024 in both patients aged 60–74 years who are at increased risk for severe RSV disease and all patients aged ≥75 years, among HCPs whose workplaces stock RSV vaccines (N = 255).^c^ Note: Bold values indicate statistical significance (*P* ≤ 0.05) based on Wald chi-square tests. ^a^HCP type, gender, years of experience, percentage of adult patients seen at practice who are aged ≥60 years, percentage of adult patients seen at practice who are non-White, percentage of adult patients seen at practice who are Hispanic/Latino, US Census region, rurality, percentage of adult patients seen at practice who are insured with commercial/private insurance, percentage of adult patients seen at practice who are insured with Medicaid, number of adult patients seen per week with respiratory infections during the respiratory infection season, familiarity with RSV disease, and stocking of RSV vaccines at workplace were considered for potential model inclusion. ^b^HCP type, gender, years of experience, percentage of adult patients seen at practice who are aged ≥60 years, percentage of adult patients seen at practice who are non-White, percentage of adult patients seen at practice who are Hispanic/Latino, US Census region, rurality, percentage of adult patients seen at practice who are insured with commercial/private insurance, percentage of adult patients seen at practice who are insured with Medicaid, number of adult patients seen per week with respiratory infections during the respiratory infection season, familiarity with RSV disease, stocking of RSV vaccines at workplace, perceived patient receptivity to RSV vaccine recommendations, knowledge of RSV vaccine efficacy, use of supports to inform RSV vaccine recommendations in adult patients aged ≥60 years, knowledge about FDA-approved indications for RSV vaccines for patients aged ≥60 years, knowledge about ACIP RSV vaccine recommendations for patients aged ≥60 years, and perception that patient age (≥60 years) is a risk factor for severe RSV disease were considered for potential model inclusion. ^c^HCP type, gender, years of experience, percentage of adult patients seen at practice who are aged ≥60 years, percentage of adult patients seen at practice who are non-White, percentage of adult patients seen at practice who are Hispanic/Latino, US Census region, rurality, percentage of adult patients seen at practice who are insured with commercial/private insurance, percentage of adult patients seen at practice who are insured with Medicaid, number of adult patients seen per week with respiratory infections during the respiratory infection season, perceived patient receptivity to RSV vaccine recommendations, knowledge of RSV vaccine efficacy, use of supports to inform RSV vaccine recommendations in adult patients aged ≥60 years, knowledge about FDA-approved indications for RSV vaccines for patients aged ≥60 years, knowledge about ACIP RSV vaccine recommendations for patients aged ≥60 years, and perception that patient age (≥60 years) is a risk factor for severe RSV disease were considered for potential model inclusion. Abbreviations: ACIP, Advisory Committee on Immunization Practices; CI, confidence interval; HCP, healthcare professional; NP, nurse practitioner; OR, odds ratio; PA, physician assistant; PCP, primary care physician; RSV, respiratory syncytial virus; US, United States.

Among the 255 HCPs whose workplaces stocked RSV vaccines, those who reported administering an RSV vaccine to ≤10% of their patients aged 60–74 years at increased risk for severe RSV disease and ≤15% of their patients aged ≥75 years were within the bottom quartile for these two patient age groups. The odds of being in this bottom quartile were higher among NPs and PAs, compared with PCPs (6.91 [1.93–24.76]). Moreover, the odds of being in the bottom quartile were higher among HCPs reporting that ≥30% of their patients were on Medicaid versus ≤5% on Medicaid (3.52 [1.11–11.19]) and among HCPs who were not fully knowledgeable about ACIP recommendations (2.59 [1.22–5.52]; Fig 6).

## Discussion

This study assessed adult RSV disease- and vaccine-related KAP among 700 US HCPs (PCPs, specialist physicians, NPs and PAs, and pharmacists), including an exploratory analysis of characteristics associated with RSV vaccination knowledge and practices.

Most HCPs reported being familiar with RSV disease, with almost all (98.6%) indicating at least some self-reported knowledge. HCPs generally associated increased age as a risk factor for severe RSV disease, aligning with literature regarding older adult vulnerability to respiratory infections [1]. However, potential knowledge gaps were observed among HCPs regarding RSV disease and RSV vaccination, including risk factors for severe RSV disease, FDA-approved indications, and ACIP recommendations for RSV vaccines. Certain risk factors for severe RSV disease, indicated by ACIP recommendations [18], were more readily recognized by HCPs than others. For instance, immunocompromised status was identified as a high risk factor for severe RSV disease by 88.0% of HCPs, compared with 33.3% classifying chronic hematologic disorders as the same risk level. Recognizing that risk factors for severe RSV disease may vary in strength of their clinical evidence [19], this risk stratification of medical conditions nonetheless indicates that HCPs may not be identifying all vaccine-eligible adults with qualifying yet potentially lesser-recognized risk factors for severe RSV disease. In the present survey, 42.3% of HCPs reported using clinical decision support systems to inform their RSV vaccine recommendations among adults aged ≥60 years; expanding the use of such systems, including electronic medical record reminders, may help to bridge this potential knowledge and practice gap of identifying risk factors for severe RSV disease among their patients.

By HCP type, pharmacists reported the lowest familiarity with RSV disease, highlighting an opportunity to enhance knowledge of RSV among this group of key vaccinators. Because RSV vaccines are covered under Medicare Part D [20], most are being administered in pharmacies [21], which can more readily submit claims for reimbursement compared with HCP offices [22,23]. This discrepancy likely contributes to the higher percentage of pharmacists (70.5%) reporting that their workplaces stock RSV vaccines, compared with other HCP types (17.6–29.6%). Moreover, considering the limited awareness and knowledge of RSV among the general US adult population [24], pharmacists are well-positioned to assume a crucial role in educating patients about RSV disease and prevention. Strengthening pharmacists’ knowledge of RSV disease may therefore help to improve vaccine access and uptake, especially considering previous research that identified pharmacies and HCP offices as convenient vaccination locations for adults in the US [25]; further research may help quantify this potential benefit.

Many HCPs either misidentified or were unsure about FDA-approved indications (72.9%) and ACIP recommendations (83.8%) for RSV vaccination across all age groups. However, RSV vaccination knowledge and awareness appears to be improving over time. In a survey conducted in November 2023, 24.9% of HCPs were unaware that any adult RSV vaccines were FDA-approved [11]; in the present survey, nearly all HCPs were aware that RSV vaccines were FDA-approved for use in adults. While this trend suggests growing awareness of the availability of adult RSV vaccines, continued education is needed to ensure HCPs are accurately informed, especially as the present survey identified that 60.1% of HCPs reported using ACIP recommendations to inform decisions regarding RSV vaccination with adult patients. In addition, most HCPs (77.7%) either underestimated or were unsure about RSV vaccine efficacy being ≥75%. This knowledge gap may adversely impact vaccination practices, as 10.3% of HCPs expressed their own concerns about vaccine effectiveness and 41.7% noted that patients had concerns about vaccine effectiveness.

Following the completion of data collection for the present study in January 2025, the ACIP expanded their recommendation in June 2025 to include adults aged 50–59 years at increased risk for severe RSV disease [10]. This change aligns with the current survey results, where 84.1% of HCPs reported that it would be beneficial or very beneficial to include at-risk adults aged 50–59 years in ACIP recommendations. Given that insurance providers are currently required to cover ACIP-recommended vaccines [20], and that pharmacists are authorized to administer only ACIP-recommended vaccines in some states [26], lack of official recommendations can create financial barriers that could discourage HCPs from recommending vaccines [27], in turn limiting patient access. In the current survey, over one-third of HCPs identified lack of insurance coverage as a potential barrier to RSV vaccination. Additionally, 34.0% of HCPs responded that their workplace requires an ACIP recommendation to be able to vaccinate, underscoring the importance of comprehensive, up-to-date clinical guidelines. The recent expansion of ACIP recommendations may therefore help address this concern and support greater RSV vaccination uptake.

Despite 84.1% of HCPs reporting that they recommend RSV vaccination to their eligible adult patients, these recommendations may not be applied consistently, potentially limiting access and contributing to disparities in access. RSV vaccination uptake from the first two seasons of vaccine availability remained relatively limited, with disparities identified by race and ethnicity and other social determinants of health [14], again highlighting a potential opportunity for the use of clinical decision support tools in supporting more equitable access to vaccination. Age-based recommendations for adults aged 60–74 years may also be more practical than risk-based recommendations in real-world settings, particularly for pharmacists who often do not have access to patients’ full clinical histories [28].

Although only 36.4% of HCPs reported their workplace stocked RSV vaccines, most (80.3%) believed that doing so would be beneficial for ensuring vaccination. When vaccines were not available onsite, 49.4% of HCPs estimated that less than half of their adult patients would follow through on their recommendations, suggesting the importance of onsite vaccine availability, as well as communication of onsite vaccine availability by workplaces to all practicing HCPs, in improving uptake following HCP recommendation. As identified in the regression analyses, HCPs whose workplaces stocked RSV vaccines were more likely to be knowledgeable of adult RSV vaccine ACIP recommendations. This finding indicates a potential opportunity to simultaneously bolster both vaccine-related knowledge and practices among HCPs and highlights the potential benefits of institutional policies that support consistently stocking RSV vaccines in healthcare settings. Furthermore, given the prevalence of vaccine hesitancy and fatigue in the US [29], offering RSV vaccination at the time and location of recommendation may enhance uptake by eliminating the need for individuals to schedule an additional appointment and travel to a secondary location for immunization.

Shared clinical decision-making, clinical judgment, and patient inquiries were reported as key approaches used to inform RSV vaccination recommendations in this study. Meanwhile, HCPs identified patients’ vaccine refusal/hesitancy, lack of RSV awareness, and vaccine safety concerns as common barriers to recommending and administering RSV vaccines. The responsibility of discussing and recommending RSV vaccination is largely dependent on HCPs, given the limited awareness of RSV among adults at increased risk for severe RSV disease as identified in a 2024–2025 survey [30]. While approximately one-third of these surveyed adults responded they would be somewhat to very unlikely to get an RSV vaccine within the next year even if an HCP recommended it, the most commonly reported barriers in this prior study were not knowing enough about the vaccine, having concerns about the safety and effectiveness of the vaccine, and not thinking they were at risk for RSV [30]. These findings suggest an opportunity for HCPs to address vaccine hesitancy with patients using methods discussed in previous studies, such as offering evidence-based information about risk for RSV disease and vaccine safety and effectiveness, engaging in open dialogue, building trust, and understanding relevant cultural and social norms [31,32].

Multivariable analyses showed that HCP type, RSV disease familiarity, and workplace practices significantly influenced vaccination knowledge and practices. By HCP type, compared with PCPs, NPs and PAs were less likely to be informed about ACIP recommendations, while pharmacists were more likely to be informed. HCPs who reported having RSV vaccines stocked at their workplace were also more likely to be informed about ACIP recommendations, compared with those who reported their workplace did not have RSV vaccines stocked. Greater familiarity with RSV disease, stocking vaccines onsite, using clinical decision support tools, and recognizing age as a risk factor were all associated with increased RSV vaccination recommendations. Moreover, knowledge of ACIP recommendations for patients aged ≥60 years was associated with increased vaccine administration among HCPs whose workplace stocked RSV vaccines. These findings are consistent with prior research indicating that limited vaccine knowledge and vaccine hesitancy among HCPs can impede vaccine recommendations [33], and support the need for targeted interventions to address potential knowledge gaps and misperceptions.

Overall, findings from this study suggest that potential knowledge gaps regarding RSV disease and vaccination remain among HCPs, despite increases in RSV disease and vaccination knowledge over time [11]. The trends observed indicate growing familiarity with RSV vaccines and evolving recommendations, reflecting progress in HCP education and vaccination implementation efforts. Nonetheless, continued efforts to expand education and awareness are still needed to ensure consistent practices across all HCP types and access to RSV vaccination among adults at increased risk for severe RSV disease. Given emerging evidence suggesting similar knowledge gaps globally [12], further research is warranted to contextualize RSV-related KAP among HCPs beyond the US to inform tailored, country-specific interventions.

### Limitations

The survey relied on self-reported data; thus, responses could not be externally verified and may have been subject to social desirability or recall bias. However, survey questions were developed to minimize the likelihood of these biases (e.g., by focusing questions on HCPs’ experience in a given week or during a specific time). Similarly, it is possible that HCPs relied on other resources for knowledge-check questions, though survey participants were not allowed to change answers to previous questions as a mitigating measure.

As this study was cross-sectional, the results do not reflect changes over time. The survey was also fielded during a time of recent and evolving FDA-approved indications and ACIP recommendations, and prior to the most recent recommendation in June 2025 to include adults aged 50–59 years at increased risk for severe RSV disease [10,18]. The effect of this most recent recommendation is unknown and warrants further investigation.

Additionally, as with all survey data, errors that may result in bias related to respondent sampling include coverage bias, selection and sampling bias, and nonresponse bias. The survey also did not include all HCP types (e.g., nurses and other specialist physicians were excluded) and may not be fully representative of US HCP demographics (e.g., only 13.9% of HCPs reported working in a rural area). Thus, it is possible that generalizability of these findings to the broader population of HCPs is limited, though this study was designed to be descriptive in nature.

## Conclusions

Results from this 2024–2025 RSV season survey indicate that RSV-related knowledge and practices among US HCPs may be improving over time, although gaps still exist. Specifically, knowledge gaps were identified related to risk factors for severe RSV disease, suggesting that HCPs may not be consistently identifying all vaccine-eligible adults, particularly those with qualifying but lesser-recognized risk factors; additional knowledge gaps were observed regarding FDA-approved indications and ACIP recommendations for RSV vaccination. Potential practice gaps were also identified in relation to recommending RSV vaccination consistently among all eligible adults. HCPs play a critical role in implementing adult RSV vaccine recommendations; therefore, findings from this study can be used to tailor HCP and, in turn, patient education efforts, as well as encourage onsite stocking of vaccines and use of clinical decision support tools at HCP offices and pharmacies, and ultimately ensure eligible populations are able to access RSV vaccination.

## Supporting information

S1 Fig Study selection flowchart. Abbreviations: HCP, healthcare professional; NP, nurse practitioner; PA, physician assistant; PCP, primary care physician.
(TIF)

S1 Table Additional HCP sample characteristics, overall and by HCP type. (DOCX)

S2 Fig Perceptions of risk factors for severe RSV disease among HCPs. A) Patient age. B) Health conditions/patient characteristics. Note: S2a Fig presents results for the question: “For the age groups below, to what extent do you feel that age alone is a risk factor for severe RSV disease?” S2b Fig presents results for the question: “For the health conditions/patient characteristics below, to what extent is each item a risk factor for severe RSV disease among adult patients?” ^a^Including chronic obstructive pulmonary disease, emphysema, asthma, interstitial lung disease, or cystic fibrosis. ^b^Including poststroke dysphagia, amyotrophic lateral sclerosis, or muscular dystrophy. ^c^Including heart failure, coronary artery disease, or congenital heart disease (excluding isolated hypertension). ^d^Including diabetes mellitus complicated by chronic kidney disease, neuropathy, retinopathy, or other end-organ damage, or requiring treatment with insulin or SGLT2 inhibitor. ^e^Body mass index ≥40 kg/m^2^. ^f^Including cirrhosis. ^g^Including sickle cell disease or thalassemia. Abbreviations: HCP, healthcare professional; RSV, respiratory syncytial virus; SGLT2, sodium-glucose cotransporter-2.
(TIFF)

S2 Table Additional results related to HCPs’ knowledge of RSV disease and vaccination. (DOCX)

S3 Table Additional results related to HCPs’ RSV vaccination attitudes. (DOCX)

S4 Table Additional results related to HCPs’ RSV testing and vaccination practices. (DOCX)

S5 Table Additional results related to HCPs’ attitudes and practices related to vaccination in general. (DOCX)

S1 File Appendix A. Study screener and questionnaire. (DOCX)
