# Supplementary figures and images for: US healthcare professionals’ knowledge, attitudes, and practices regarding RSV disease and vaccination in adults during the 2024–2025 RSV season
Source: PLoS One. 2026 Jul 22;21(7):e0353266. doi: 10.1371/journal.pone.0353266 (PMC13390937; doi:10.1371/journal.pone.0353266)

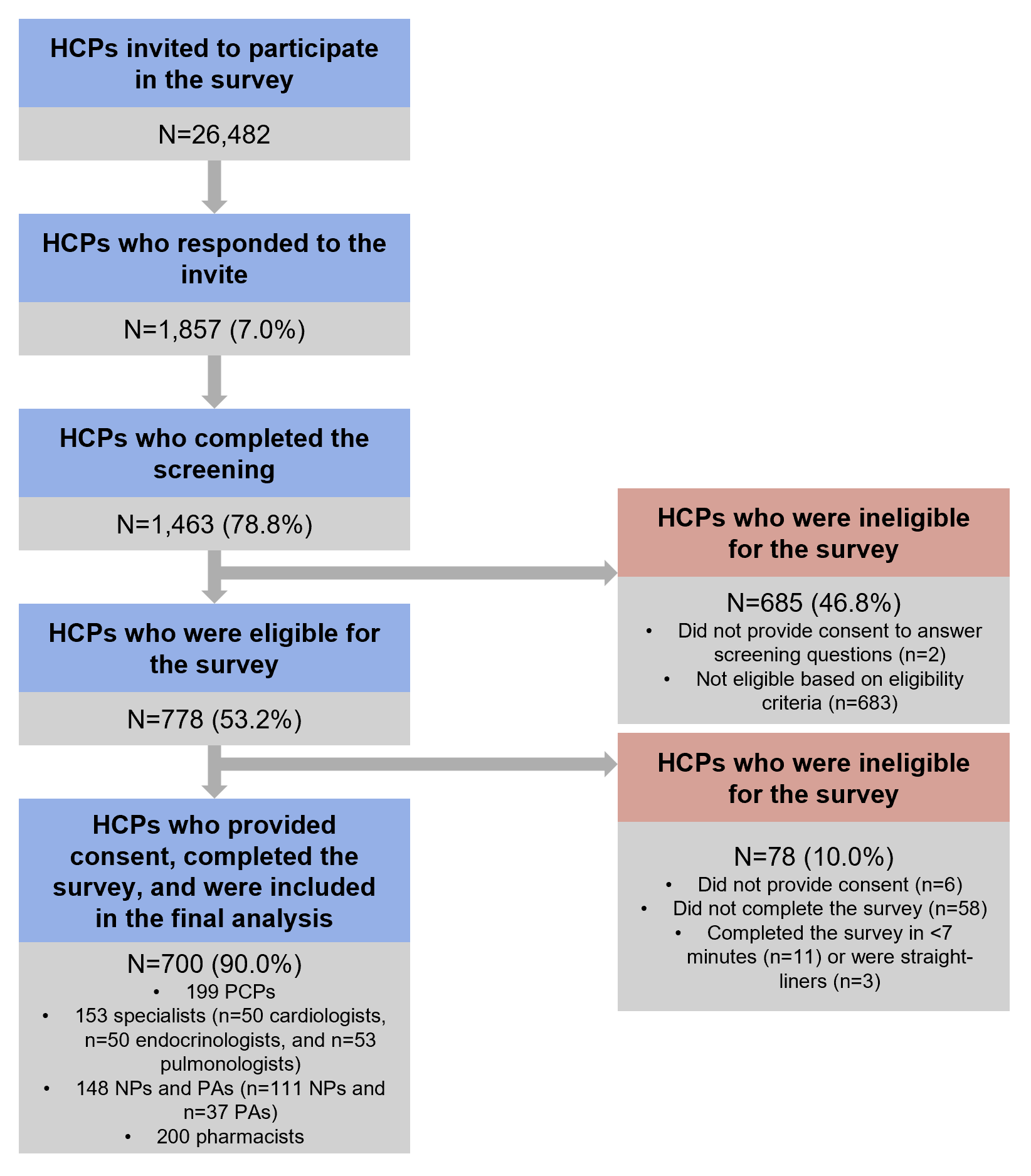

Supplement: S1 Fig — Abbreviations: HCP, healthcare professional; NP, nurse practitioner; PA, physician assistant; PCP, primary care physician. (TIF) [file pone.0353266.s001.tif]

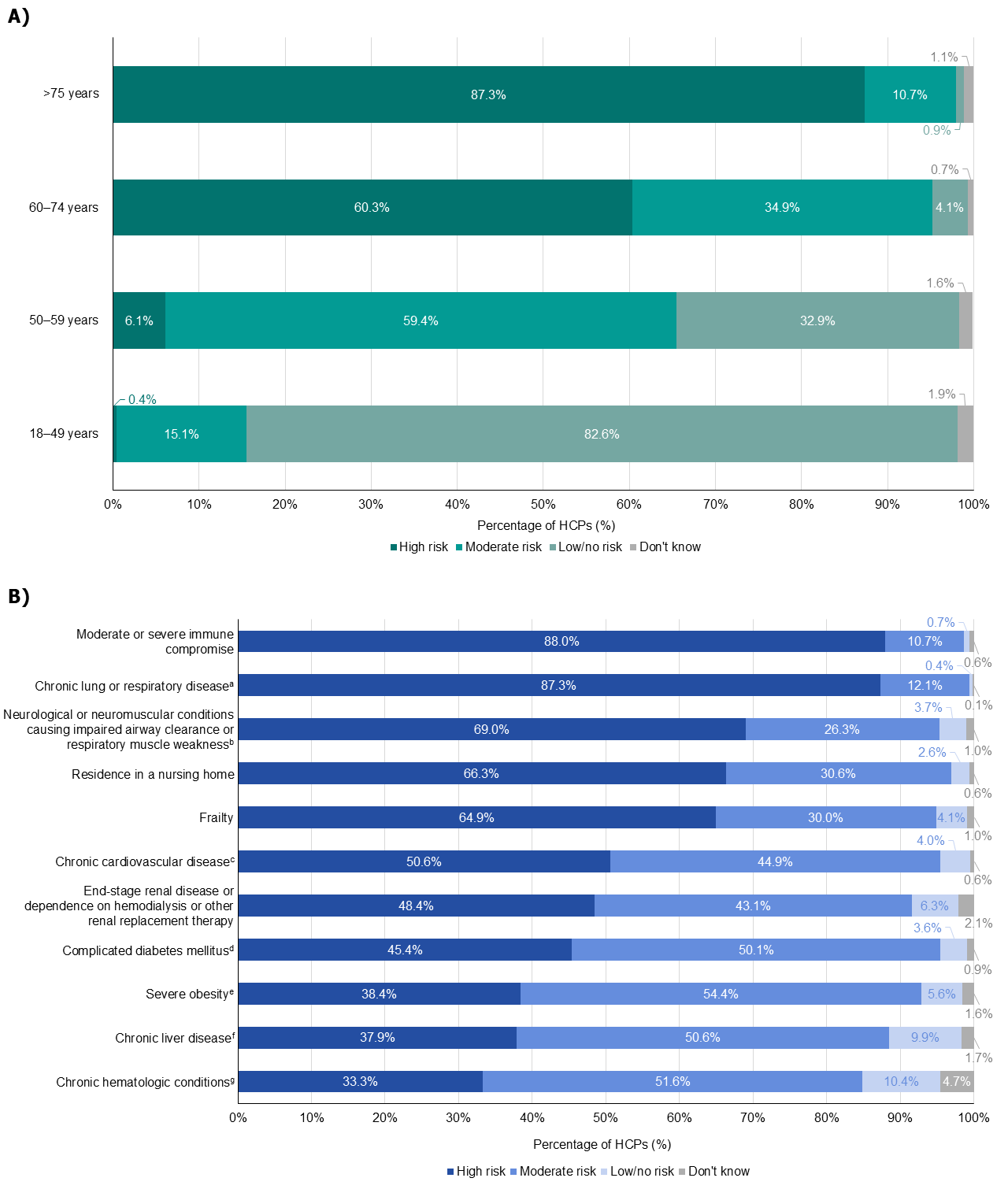

Supplement: S2 Fig — A) Patient age. B) Health conditions/patient characteristics. Note: S2a Fig presents results for the question: “For the age groups below, to what extent do you feel that age alone is a risk factor for severe RSV disease?” S2b Fig presents results for the question: “For the health conditions/patient characteristics below, to what extent is each item a risk factor for severe RSV disease among adult patients?” aIncluding chronic obstructive pulmonary disease, emphysema, asthma, interstitial lung disease, or cystic fibrosis. bIncluding poststroke dysphagia, amyotrophic lateral sclerosis, or muscular dystrophy. cIncluding heart failure, coronary artery disease, or congenital heart disease (excluding isolated hypertension). dIncluding diabetes mellitus complicated by chronic kidney disease, neuropathy, retinopathy, or other end-organ damage, or requiring treatment with insulin or SGLT2 inhibitor. eBody mass index ≥40 kg/m2. fIncluding cirrhosis. gIncluding sickle cell disease or thalassemia. Abbreviations: HCP, healthcare professional; RSV, respiratory syncytial virus; SGLT2, sodium-glucose cotransporter-2. (TIFF) [file pone.0353266.s003.tiff]
