# Supplementary material for: US healthcare professionals’ knowledge, attitudes, and practices regarding RSV disease and vaccination in adults during the 2024–2025 RSV season
Source: PLoS One. 2026 Jul 22;21(7):e0353266. doi: 10.1371/journal.pone.0353266 (PMC13390937; doi:10.1371/journal.pone.0353266)
Supplement: S1 Table — (DOCX) [file pone.0353266.s002.docx]

**S1 Table.** Additional HCP sample characteristics, overall and by HCP type

|  | **Overall**  **(N=700)** | **PCPs**  **(N=199)** | **Specialists**  **(N=153)** | **NPs and PAs**  **(N=148)** | **Pharmacists**  **(N=200)** |
| --- | --- | --- | --- | --- | --- |
| Primary work environment (non-pharmacists), n (%) | | | | | |
|  | N=500 | N=199 | N=153 | N=148 | N/A |
| Private practice, solo | 67 (13.4) | 44 (22.1) | 10 (6.5) | 13 (8.8) | N/A |
| Private practice, single specialty group | 127 (25.4) | 47 (23.6) | 40 (26.1) | 40 (27.0) | N/A |
| Private practice, multispecialty group | 105 (21.0) | 42 (21.1) | 38 (24.8) | 25 (16.9) | N/A |
| Managed care or health maintenance organization (HMO) practice | 17 (3.4) | 6 (3.0) | 5 (3.3) | 6 (4.1) | N/A |
| Outpatient clinic (e.g., ambulatory care center, urgent care center) | 104 (20.8) | 37 (18.6) | 11 (7.2) | 56 (37.8) | N/A |
| Academic medical center | 80 (16.0) | 23 (11.6) | 49 (32.0) | 8 (5.4) | N/A |
| Primary work environment (pharmacists), n (%) | | | | | |
|  | N/A | N/A | N/A | N/A | N=200 |
| Large pharmacy chain (e.g., CVS, Walgreens, Rite Aid) | N/A | N/A | N/A | N/A | 80 (40.0) |
| Grocery store/chain (e.g., Albertson's, Publix, HEB, Kroger, Wegmans) | N/A | N/A | N/A | N/A | 42 (21.0) |
| Mass merchant (e.g., Walmart, Costco) | N/A | N/A | N/A | N/A | 28 (14.0) |
| Regional pharmacy chain (e.g., Kinney Drugs, Thrifty White) | N/A | N/A | N/A | N/A | 2 (1.0) |
| Independent pharmacy | N/A | N/A | N/A | N/A | 48 (24.0) |
| Hours worked in primary medical profession per week, n (%) | | | | | |
| 21–30 hours | 73 (10.4) | 20 (10.1) | 10 (6.5) | 24 (16.2) | 19 (9.5) |
| 31–40 hours | 321 (45.9) | 88 (44.2) | 51 (33.3) | 93 (62.8) | 89 (44.5) |
| >40 hours | 306 (43.7) | 91 (45.7) | 92 (60.1) | 31 (20.9) | 92 (46.0) |
| Estimated percentage of adult patients (aged ≥18 years) seen in the workplace that fall under each race category, mean (SD) | | | | | |
| Asian | 9.7 (11.1) | 10.0 (10.0) | 9.8 (10.1) | 7.2 (7.5) | 11.2 (14.3) |
| Black or African American | 19.6 (15.8) | 16.6 (14.6) | 21.2 (13.8) | 22.5 (17.5) | 19.3 (16.7) |
| White | 58.6 (22.1) | 60.6 (22.2) | 60.0 (18.2) | 56.0 (23.6) | 57.5 (23.4) |
| Another race not listed | 12.1 (15.3) | 12.8 (16.2) | 9.0 (11.0) | 14.3 (18.0) | 12.0 (14.8) |
| Percentage of adult patients (aged ≥18 years) seen in the workplace that are Hispanic, Latina(o), Latine, or Latinx, mean (SD) | | | | | |
|  | 19.9 (19.1) | 19.5 (19.0) | 18.2 (16.7) | 22.7 (21.2) | 19.5 (19.0) |
| Approximate percentage of adult patients (aged ≥18 years) seen in the workplace who have each of the following types of health insurance, mean, (SD) | | | | | |
| Commercial/private insurance | 40.5 (21.5) | 47.8 (23.0) | 42.9 (17.3) | 35.2 (25.2) | 35.4 (17.2) |
| Medicaid | 18.4 (17.1) | 13.0 (16.4) | 13.5 (11.1) | 23.8 (22.0) | 23.7 (14.7) |
| Medicare | 31.5 (17.0) | 30.5 (18.4) | 34.8 (15.0) | 29.6 (21.3) | 31.4 (12.8) |
| Other health insurance | 4.3 (9.5) | 4.8 (9.5) | 4.7 (9.2) | 5.1 (13.7) | 3.0 (4.6) |
| No health insurance | 5.3 (9.6) | 4.0 (8.0) | 4.1 (7.3) | 6.4 (14.1) | 6.5 (8.3) |
| Approximate percentage of patients personally evaluated and/or treated OR interacted with in each of the following age groups, mean, (SD) | | | | | |
| <18 years | 9.2 (12.0) | 9.2 (12.1) | 4.4 (14.7) | 8.6 (11.8) | 13.4 (7.6) |
| 18–59 years | 45.5 (17.1) | 47.7 (18.0) | 45.6 (15.1) | 49.4 (20.8) | 40.4 (13.0) |
| ≥60 years | 45.1 (18.2) | 43.2 (19.1) | 50.0 (16.7) | 42.0 (22.6) | 45.7 (13.5) |
| During an average week, about how many adult patients aged ≥18 years do you personally evaluate and/or treat OR interact with?^a^ Mean, (SD) | | | | | |
|  | 109.8 (93.9) | 91.4 (59.0) | 90.0 (73.1) | 79.7 (65.9) | 165.5 (125.5) |

^a^“Personally evaluate and/or treat” was used if HCP indicated their primary medical profession as a PCP, specialist, NP, or PA. “Interact with” was used if HCP indicated their primary medical profession as a pharmacist. A maximum value of 250 was originally set on this item during the soft launch of the survey, and n=4/38 participants entered the maximum of 250; it is possible that their true response was >250. The maximum was increased to 500 for the remainder of the sample; of these, n=13/662 entered the maximum value of 500. Abbreviations: HCP, healthcare professional; HMO, health maintenance organization; N/A, not applicable; NP, nurse practitioner; PA, physician assistant; PCP, primary care physician; SD, standard deviation.
