# Supplementary material for: US healthcare professionals’ knowledge, attitudes, and practices regarding RSV disease and vaccination in adults during the 2024–2025 RSV season
Source: PLoS One. 2026 Jul 22;21(7):e0353266. doi: 10.1371/journal.pone.0353266 (PMC13390937; doi:10.1371/journal.pone.0353266)
Supplement: S2 Table — (DOCX) [file pone.0353266.s004.docx]

**S2 Table.** Additional results related to HCPs’ knowledge of RSV disease and vaccination

|  | **Overall** | **PCPs** | **Specialists** | **NPs and PAs** | **Pharmacists** |
| --- | --- | --- | --- | --- | --- |
|  | **(N=700)** | **(N=199)** | **(N=153)** | **(N=148)** | **(N=200)** |
| Correctly responded that 3 RSV vaccines are currently FDA-approved for use in adults, n (%) | 176 (25.1) | 41 (20.6) | 37 (24.2) | 21 (14.2) | 77 (38.5) |
| Correctly identified FDA approvals for RSV vaccines for non-pregnant adult groups, n (%) | | | | | |
| Adults aged 18–49 years who are at increased risk for severe RSV disease* | 315 (45.0) | 95 (47.7) | 67 (43.8) | 67 (45.3) | 86 (43.0) |
| Adults aged 50–59 years who are at increased risk for severe RSV disease* | 368 (52.6) | 111 (55.8) | 78 (51.0) | 83 (56.1) | 96 (48.0) |
| All adults aged 60–74 years* | 424 (60.6) | 125 (62.8) | 94 (61.4) | 101 (68.2) | 104 (52.0) |
| All adults aged ≥75 years* | 598 (85.4) | 174 (87.4) | 125 (81.7) | 118 (79.7) | 181 (90.5) |
| Correct about FDA approvals across all age groups | 190 (27.1) | 59 (29.6) | 42 (27.5) | 48 (32.4) | 41 (20.5) |
| Correctly identified ACIP recommendation for RSV vaccines for non-pregnant adult groups, n (%) | | | | | |
|  | N=698 | N=198 | N=152 | N=148 | N=200 |
| Not ACIP-recommended for any adults aged 18–49 years* | 224 (32.1) | 63 (31.8) | 42 (27.6) | 30 (20.3) | 89 (44.5) |
| Not ACIP-recommended for any adults aged 50–59 years*^a^ | 148 (21.2) | 36 (18.2) | 26 (17.1) | 16 (10.8) | 70 (35.0) |
| ACIP-recommended among adults aged 60–74 years who are at increased risk for severe RSV disease* | 243 (34.8) | 65 (32.8) | 41 (27.0) | 32 (21.6) | 105 (52.5) |
| ACIP-recommended among adults aged ≥75 years* | 562 (80.5) | 161 (81.3) | 114 (75.0) | 107 (72.3) | 180 (90.0) |
| Correct about ACIP recommendations across all age groups | 113 (16.2) | 25 (12.6) | 18 (11.8) | 7 (4.7) | 63 (31.5) |
| To your knowledge, what percentage of RSV lower respiratory tract disease cases would the currently available RSV vaccines prevent in adults aged 60 years and older during the first season after vaccination (i.e., what is the vaccine efficacy)? (n, %) | | | | | |
| <35% | 38 (5.4) | 14 (7.0) | 5 (3.3) | 8 (5.4) | 11 (5.5) |
| 35–54% | 143 (20.4) | 47 (23.6) | 30 (19.6) | 31 (20.9) | 35 (17.5) |
| 55–74% | 210 (30.0) | 58 (29.1) | 48 (31.4) | 37 (25.0) | 67 (33.5) |
| ≥75%* | 156 (22.3) | 40 (20.1) | 35 (22.9) | 34 (23.0) | 47 (23.5) |
| Don’t know | 153 (21.9) | 40 (20.1) | 35 (22.9) | 38 (25.7) | 40 (20.0) |
| Number of adult patients with respiratory infections seen during an average week within the respiratory infection season, n (%) | | | | | |
| None | 20 (2.9) | 0 (0.0) | 13 (8.5) | 7 (4.7) | 0 (0.0) |
| <10 | 147 (21.0) | 31 (15.6) | 66 (43.1) | 34 (23.0) | 16 (8.0) |
| 10–25 | 322 (46.0) | 124 (62.3) | 53 (34.6) | 64 (43.2) | 81 (40.5) |
| 26–50 | 146 (20.9) | 37 (18.6) | 17 (11.1) | 27 (18.2) | 65 (32.5) |
| >50 | 65 (9.3) | 7 (3.5) | 4 (2.6) | 16 (10.8) | 38 (19.0) |
| Percentage of adult patients with respiratory infections during the respiratory infection season that HCPs suspect have RSV, n (%) | | | | | |
|  | N=679 | N=198 | N=140 | N=141 | N=200 |
| None | 9 (1.3) | 0 (0.0) | 1 (0.7) | 2 (1.4) | 6 (3.0) |
| <5% | 186 (27.4) | 55 (27.8) | 28 (20.0) | 52 (36.9) | 51 (25.5) |
| 5–14% | 226 (33.3) | 78 (39.4) | 48 (34.3) | 47 (33.3) | 53 (26.5) |
| 15–24% | 134 (19.7) | 44 (22.2) | 32 (22.9) | 19 (13.5) | 39 (19.5) |
| 25–49% | 53 (7.8) | 15 (7.6) | 16 (11.4) | 6 (4.3) | 16 (8.0) |
| ≥50% | 13 (1.9) | 1 (0.5) | 5 (3.6) | 3 (2.1) | 4 (2.0) |
| Don’t know | 58 (8.5) | 5 (2.5) | 10 (7.1) | 12 (8.5) | 31 (15.5) |
| Correctly identified the percentage of medically attended RSV cases among adults aged ≥60 years that result in hospitalization (5–14%*), n (%) | 260 (37.1) | 76 (38.2) | 57 (37.3) | 51 (34.5) | 76 (38.0) |
| Correctly identified the percentage of hospitalized RSV cases among adults aged ≥60 years that experience in-hospital death (4–8%*), n (%) | 258 (36.9) | 62 (31.3) | 66 (43.1) | 62 (41.9) | 68 (34.0) |
| Agreement or disagreement with the statement “RSV disease impacts patients’ underlying conditions (e.g., COPD, asthma)” (n, %) | | | | | |
| Strongly agree | 383 (54.7) | 106 (53.3) | 82 (53.6) | 90 (60.8) | 105 (52.5) |
| Agree | 260 (37.1) | 76 (38.2) | 58 (37.9) | 49 (33.1) | 77 (38.5) |
| Disagree | 5 (0.7) | 1 (0.5) | 2 (1.3) | 0 (0.0) | 2 (1.0) |
| Strongly disagree | 50 (7.1) | 14 (7.0) | 11 (7.2) | 9 (6.1) | 16 (8.0) |
| Don’t know | 2 (0.3) | 2 (1.0) | 0 (0.0) | 0 (0.0) | 0 (0.0) |

*Denotes correct response. ^a^Correct at the time of survey administration. In June 2025, ACIP recommendations for RSV vaccination were updated to include adults aged 50–59 years who are at increased risk of severe RSV disease. Abbreviations: ACIP, Advisory Committee on Immunization Practices; COPD, chronic obstructive pulmonary disease; FDA, Food and Drug Administration; HCP, healthcare professional; NP, nurse practitioner; PA, physician assistant; PCP, primary care physician; RSV, respiratory syncytial virus.
