# Supplementary material for: US healthcare professionals’ knowledge, attitudes, and practices regarding RSV disease and vaccination in adults during the 2024–2025 RSV season
Source: PLoS One. 2026 Jul 22;21(7):e0353266. doi: 10.1371/journal.pone.0353266 (PMC13390937; doi:10.1371/journal.pone.0353266)
Supplement: S3 Table — (DOCX) [file pone.0353266.s005.docx]

**S3 Table.** Additional results related to HCPs’ RSV vaccination attitudes

|  | **Overall** | **PCPs** | **Specialists** | **NPs and PAs** | **Pharmacists** |
| --- | --- | --- | --- | --- | --- |
|  | **(N=700)** | **(N=199)** | **(N=153)** | **(N=148)** | **(N=200)** |
| To what extent do you agree that stocking RSV vaccine(s) for administration at your workplace helps/would help to ensure at-risk individuals get vaccinated? (n, %) | | | | | |
| Strongly agree | 269 (38.4) | 65 (32.7) | 42 (27.5) | 42 (28.4) | 120 (60.0) |
| Agree | 293 (41.9) | 92 (46.2) | 70 (45.8) | 66 (44.6) | 65 (32.5) |
| Neither agree nor disagree | 121 (17.3) | 31 (15.6) | 40 (26.1) | 37 (25.0) | 13 (6.5) |
| Disagree | 10 (1.4) | 6 (3.0) | 1 (0.7) | 1 (0.7) | 2 (1.0) |
| Strongly disagree | 7 (1.0) | 5 (2.5) | 0 (0.0) | 2 (1.4) | 0 (0.0) |
| Among patients to whom you've recommended RSV vaccination, what percentage in each of the following age groups do you think have followed through and gotten vaccinated? Mean (SD) | | | | | |
| 50–59 years | N=273  35.5 (26.1) | N=93  40.7 (26.6) | N=63  42.1 (25.3) | N=43  24.3 (23.9) | N=74  29.9 (24.4) |
| 60–74 years | N=451  48.9 (26.2) | N=130  51.5 (26.9) | N=89  56.8 (24.1) | N=80  40.9 (25.6) | N=152  46.3 (25.7) |
| ≥75 years | N=465  59.3 (27.1) | N=133  62.2 (26.8) | N=93  65.9 (23.9) | N=81  50.6 (29.2) | N=158  57.6 (26.8) |
| To what extent do you agree with the following statements regarding RSV vaccinations? (n, %) | | | | | |
| “Adults aged 50–59 years who are at increased risk of severe RSV disease should be included in ACIP recommendations for RSV vaccination.”^a^ | N=699 | N=199 | N=152 | N=148 | N=200 |
| Strongly agree | 176 (25.2) | 62 (31.2) | 43 (28.3) | 30 (20.3) | 41 (20.5) |
| Agree | 433 (61.9) | 115 (57.8) | 87 (57.2) | 99 (66.9) | 132 (66.0) |
| Disagree | 24 (3.4) | 5 (2.5) | 4 (2.6) | 4 (2.7) | 11 (5.5) |
| Strongly disagree | 5 (0.7) | 2 (1.0) | 1 (0.7) | 1 (0.7) | 1 (0.5) |
| Don't know | 61 (8.7) | 15 (7.5) | 17 (11.2) | 14 (9.5) | 15 (7.5) |
| Missing | 1 | 0 | 1 | 0 | 0 |
| “The benefits of RSV vaccination outweigh the potential risks for adults aged 50–59 years who are at increased risk of severe RSV disease.”^a^ |  |  |  |  |  |
| Strongly agree | 189 (27.0) | 54 (27.1) | 54 (35.3) | 31 (20.9) | 50 (25.0) |
| Agree | 409 (58.4) | 118 (59.3) | 77 (50.3) | 88 (59.5) | 126 (63.0) |
| Disagree | 28 (4.0) | 7 (3.5) | 4 (2.6) | 10 (6.8) | 7 (3.5) |
| Strongly disagree | 2 (0.3) | 2 (1.0) | 0 (0.0) | 0 (0.0) | 0 (0.0) |
| Don't know | 72 (10.3) | 18 (9.0) | 18 (11.8) | 19 (12.8) | 17 (8.5) |
| “Adults aged 50–59 years who are at increased risk of severe RSV disease can get the RSV vaccine even though the ACIP has not provided a recommendation in this group.”^a^ |  |  |  |  |  |
| Strongly agree | 144 (20.6) | 54 (27.1) | 34 (22.2) | 22 (14.9) | 34 (17.0) |
| Agree | 335 (47.9) | 93 (46.7) | 78 (51.0) | 78 (52.7) | 86 (43.0) |
| Disagree | 91 (13.0) | 21 (10.6) | 12 (7.8) | 17 (11.5) | 41 (20.5) |
| Strongly disagree | 19 (2.7) | 5 (2.5) | 2 (1.3) | 0 (0.0) | 12 (6.0) |
| Don't know | 111 (15.9) | 26 (13.1) | 27 (17.6) | 31 (20.9) | 27 (13.5) |
| “I am concerned that my adult patients aged 50–59 years who are at increased risk of severe RSV disease won't be able to get the RSV vaccine because the ACIP has not provided a recommendation in this group.”^a^ |  |  |  |  |  |
| Strongly agree | 114 (16.3) | 34 (17.1) | 32 (20.9) | 22 (14.9) | 26 (13.0) |
| Agree | 365 (52.1) | 104 (52.3) | 69 (45.1) | 83 (56.1) | 109 (54.5) |
| Disagree | 103 (14.7) | 29 (14.6) | 26 (17.0) | 14 (9.5) | 34 (17.0) |
| Strongly disagree | 16 (2.3) | 7 (3.5) | 1 (0.7) | 3 (2.0) | 5 (2.5) |
| Don't know | 102 (14.6) | 25 (12.6) | 25 (16.3) | 26 (17.6) | 26 (13.0) |

^a^Following survey administration, in June 2025, ACIP recommendations were updated to include RSV vaccination among increased-risk adults aged 50–59 years. Abbreviations: ACIP, Advisory Committee on Immunization Practices; HCP, healthcare professional; NP, nurse practitioner; PA, physician assistant; PCP, primary care physician; RSV, respiratory syncytial virus; SD, standard deviation.
