# Supplementary material for: US healthcare professionals’ knowledge, attitudes, and practices regarding RSV disease and vaccination in adults during the 2024–2025 RSV season
Source: PLoS One. 2026 Jul 22;21(7):e0353266. doi: 10.1371/journal.pone.0353266 (PMC13390937; doi:10.1371/journal.pone.0353266)
Supplement: S4 Table — (DOCX) [file pone.0353266.s006.docx]

**S4 Table.** Additional results related to HCPs’ RSV testing and vaccination practices

|  | **Overall** | **PCPs** | **Specialists** | **NPs and PAs** | **Pharmacists** |
| --- | --- | --- | --- | --- | --- |
|  | **(N=700)** | **(N=199)** | **(N=153)** | **(N=148)** | **(N=200)** |
| How often do you test (or order a lab test) for RSV in your adult patients (aged 18 years and older) with respiratory infections? (n, %)^a^ | | | | | |
|  | N=500 | N=199 | N=153 | N=148 | N/A |
| Always | 22 (4.4) | 11 (5.5) | 9 (5.9) | 2 (1.4) | N/A |
| Often | 95 (19.0) | 49 (24.6) | 28 (18.3) | 18 (12.2) | N/A |
| Sometimes | 165 (33.0) | 77 (38.7) | 47 (30.7) | 41 (27.7) | N/A |
| Rarely | 132 (26.4) | 43 (21.6) | 37 (24.2) | 52 (35.1) | N/A |
| Never | 86 (17.2) | 19 (9.5) | 32 (20.9) | 35 (23.6) | N/A |
| Since September 1, 2024, please estimate the percentage of adult patients in each group listed below with whom you discussed, recommended, and/or administered RSV vaccination. Please consider only your personal interactions and not the experiences of other healthcare professionals in your workplace. Mean (SD), N=699 | | | | | |
| Discussed RSV vaccination | | | | | |
| Adults aged 50–59 years who are at increased risk | 31.2 (33.4) | 41.4 (36.7) | 31.7 (35.5) | 31.8 (33.3) | 20.4 (23.9) |
| Healthy adults aged 60–74 years | 41.5 (34.8) | 49.2 (36.1) | 38.4 (37.2) | 44.3 (37.1) | 34.1 (27.7) |
| Adults aged 60–74 years who are at increased risk | 52.8 (36.2) | 64.0 (33.9) | 51.6 (39.3) | 51.5 (38.7) | 43.4 (30.8) |
| All adults aged ≥75 years | 57.1 (37.6) | 68.5 (35.0) | 54.4 (39.9) | 53.9 (40.2) | 50.1 (33.7) |
| Recommended RSV vaccination | | | | | |
| Adults aged 50–59 years who are at increased risk | 29.8 (34.2) | 39.2 (37.6) | 34.2 (36.7) | 26.6 (32.7) | 19.6 (25.9) |
| Healthy adults aged 60–74 years | 40.5 (35.9) | 46.7 (37.3) | 43.0 (37.3) | 41.2 (37.3) | 31.8 (30.4) |
| Adults aged 60–74 years who are at increased risk | 53.7 (36.5) | 63.1 (34.6) | 54.3 (39.0) | 53.9 (38.8) | 44.0 (32.3) |
| All adults aged ≥75 years | 58.8 (37.1) | 68.0 (35.1) | 58.2 (38.3) | 56.7 (39.7) | 51.7 (34.4) |
| Personally (or someone else at my workplace) administered RSV vaccination | | | | | |
| Adults aged 50–59 years who are at increased risk | 12.2 (22.4) | 14.5 (24.7) | 8.9 (19.9) | 7.8 (19.5) | 15.8 (23.2) |
| Healthy adults aged 60–74 years | 16.8 (25.8) | 19.8 (29.1) | 10.3 (20.6) | 10.5 (23.0) | 23.5 (25.9) |
| Adults aged 60–74 years who are at increased risk | 22.2 (29.7) | 24.0 (31.8) | 16.8 (28.6) | 12.5 (26.7) | 31.8 (27.4) |
| All adults aged ≥75 years | 24.9 (31.7) | 27.7 (34.7) | 17.5 (29.1) | 13.6 (28.7) | 36.3 (28.3) |
| Reflecting on your experiences discussing RSV vaccination with patients, how often do patients in each of the following groups initiate the discussion? (n, %) | | | | | |
| 50–59 years | | | | | |
| Most of the time | 24 (3.4) | 3 (1.5) | 2 (1.3) | 5 (3.4) | 14 (7.0) |
| Often | 45 (6.4) | 14 (7.0) | 5 (3.3) | 12 (8.1) | 14 (7.0) |
| Sometimes | 181 (25.9) | 50 (25.1) | 26 (17.0) | 41 (27.7) | 64 (32.0) |
| Rarely | 381 (54.4) | 118 (59.3) | 102 (66.7) | 74 (50.0) | 87 (43.5) |
| I have not discussed RSV vaccination with this age group | 69 (9.9) | 14 (7.0) | 18 (11.8) | 16 (10.8) | 21 (10.5) |
| 60–74 years | | | | | |
| Most of the time | 52 (7.4) | 12 (6.0) | 7 (4.6) | 9 (6.1) | 24 (12.0) |
| Often | 141 (20.1) | 33 (16.6) | 22 (14.4) | 30 (20.3) | 56 (28.0) |
| Sometimes | 317 (45.3) | 90 (45.2) | 66 (43.1) | 63 (42.6) | 98 (49.0) |
| Rarely | 164 (23.4) | 61 (30.7) | 50 (32.7) | 36 (24.3) | 17 (8.5) |
| I have not discussed RSV vaccination with this age group | 26 (3.7) | 3 (1.5) | 8 (5.2) | 10 (6.8) | 5 (2.5) |
| ≥75 years | | | | | |
| Most of the time | 99 (14.1) | 22 (11.1) | 17 (11.1) | 16 (10.8) | 44 (22.0) |
| Often | 186 (26.6) | 49 (24.6) | 27 (17.6) | 35 (23.6) | 75 (37.5) |
| Sometimes | 249 (35.6) | 76 (38.2) | 60 (39.2) | 54 (36.5) | 59 (29.5) |
| Rarely | 142 (20.3) | 50 (25.1) | 42 (27.5) | 32 (21.6) | 18 (9.0) |
| I have not discussed RSV vaccination with this age group | 24 (3.4) | 2 (1.0) | 7 (4.6) | 11 (7.4) | 4 (2.0) |
| When deciding on recommending an RSV vaccination to adult patients, to what extent do you follow the CDC’s ACIP recommendations? (n, %) | | | | | |
| Always | 271 (38.7) | 54 (27.1) | 35 (22.9) | 60 (40.5) | 122 (61.0) |
| Often | 302 (43.1) | 104 (52.3) | 76 (49.7) | 58 (39.2) | 64 (32.0) |
| Sometimes | 71 (10.1) | 34 (17.1) | 16 (10.5) | 14 (9.5) | 7 (3.5) |
| Rarely | 8 (1.1) | 3 (1.5) | 1 (0.7) | 1 (0.7) | 3 (1.5) |
| Never | 2 (0.3) | 0 (0.0) | 1 (0.7) | 1 (0.7) | 0 (0.0) |
| I am not familiar with the ACIP recommendations for RSV vaccination | 46 (6.6) | 4 (2.0) | 24 (15.7) | 14 (9.5) | 4 (2.0) |
| Which of the following approaches or processes do you currently use to inform your decision-making regarding recommending RSV vaccination in adult patients aged ≥60 years? Please select all that apply. (n, %)^b^ | | | | | |
| Clinical judgment | 478 (68.3) | 138 (69.3) | 95 (62.1) | 106 (71.6) | 139 (69.5) |
| Shared clinical decision-making | 479 (68.4) | 148 (74.4) | 101 (66.0) | 100 (67.6) | 130 (65.0) |
| Clinical decision support systems (e.g., EMR reminder, immunization alerts) | 296 (42.3) | 74 (37.2) | 55 (35.9) | 82 (55.4) | 85 (42.5) |
| Treatment protocols at my workplace | 196 (28.0) | 43 (21.6) | 26 (17.0) | 45 (30.4) | 82 (41.0) |
| My patients ask about RSV vaccination | 427 (61.0) | 109 (54.8) | 77 (50.3) | 94 (63.5) | 147 (73.5) |
| A state IIS that includes a reminder/recall for vaccinations | 34 (4.9) | 4 (2.0) | 6 (3.9) | 14 (9.5) | 10 (5.0) |
| My patients complete a questionnaire in the waiting room or prior to the visit that includes the patient's history of vaccinations | 103 (14.7) | 27 (13.6) | 21 (13.7) | 15 (10.1) | 40 (20.0) |
| Training to keep updated on immunization recommendations and/or guidelines | 291 (41.6) | 72 (36.2) | 57 (37.3) | 54 (36.5) | 108 (54.0) |
| Annual immunization schedules included on the CDC website | 334 (47.7) | 102 (51.3) | 67 (43.8) | 71 (48.0) | 94 (47.0) |
| ACIP recommendations | 421 (60.1) | 126 (63.3) | 73 (47.7) | 82 (55.4) | 140 (70.0) |
| Other professional guidelines (e.g., AACE, AAFP, AANP, AAPA, ACC, ACP, APhA, CHEST) | 222 (31.7) | 71 (35.7) | 55 (35.9) | 53 (35.8) | 43 (21.5) |
| None of the above | 10 (1.4) | 0 (0.0) | 2 (1.3) | 6 (4.1) | 2 (1.0) |
| What topics do you think are important to cover when discussing RSV vaccination with patients aged ≥60 years? Please select all that apply. (n, %)^b^ | | | | | |
| Risk of getting RSV | 531 (75.9) | 148 (74.4) | 105 (68.6) | 119 (80.4) | 159 (79.5) |
| Risk for serious illness or complications from RSV | 615 (87.9) | 178 (89.4) | 128 (83.7) | 138 (93.2) | 171 (85.5) |
| Risk of death from RSV | 469 (67.0) | 133 (66.8) | 120 (78.4) | 111 (75.0) | 105 (52.5) |
| Risk of adverse side effects from vaccine | 295 (42.1) | 88 (44.2) | 68 (44.4) | 70 (47.3) | 69 (34.5) |
| Risk of transmitting RSV to others | 336 (48.0) | 94 (47.2) | 74 (48.4) | 81 (54.7) | 87 (43.5) |
| ACIP recommendations for the RSV vaccine | 302 (43.1) | 87 (43.7) | 61 (39.9) | 62 (41.9) | 92 (46.0) |
| Safety of the RSV vaccine | 430 (61.4) | 130 (65.3) | 105 (68.6) | 91 (61.5) | 104 (52.0) |
| Efficacy of the RSV vaccine | 469 (67.0) | 138 (69.3) | 108 (70.6) | 105 (70.9) | 118 (59.0) |
| Cost of the RSV vaccine | 221 (31.6) | 69 (34.7) | 42 (27.5) | 44 (29.7) | 66 (33.0) |
| My personal recommendation for the patient | 268 (38.3) | 87 (43.7) | 62 (40.5) | 48 (32.4) | 71 (35.5) |
| None of the above | 5 (0.7) | 0 (0.0) | 2 (1.3) | 2 (1.4) | 1 (0.5) |

^a^Asked if the HCP indicated their primary medical profession as a PCP, specialist physician, NP, or PA. ^b^Not mutually exclusive. Abbreviations: AACE, American Association of Clinical Endocrinology; AAFP, American Academy of Family Physicians; AANP, American Association of Nurse Practitioners; AAPA, American Academy of Physician Associates; ACC, American College of Cardiology; ACIP, Advisory Committee on Immunization Practices; ACP, American College of Physicians; APhA, American Pharmacists Association; CDC, Centers for Disease Control and Prevention; CHEST, American College of Chest Physicians; EMR, electronic medical record; HCP, healthcare professional; IIS, immunization information systems; NP, nurse practitioner; PA, physician assistant; PCP, primary care physician; RSV, respiratory syncytial virus; SD, standard deviation.
