# Supplementary material for: US healthcare professionals’ knowledge, attitudes, and practices regarding RSV disease and vaccination in adults during the 2024–2025 RSV season
Source: PLoS One. 2026 Jul 22;21(7):e0353266. doi: 10.1371/journal.pone.0353266 (PMC13390937; doi:10.1371/journal.pone.0353266)
Supplement: S5 Table — (DOCX) [file pone.0353266.s007.docx]

**S5 Table.** Additional results related to HCPs’ attitudes and practices related to vaccination in general

|  | **Overall** | **PCPs** | **Specialists** | **NPs and PAs** | **Pharmacists** |
| --- | --- | --- | --- | --- | --- |
|  | **(N=700)** | **(N=199)** | **(N=153)** | **(N=148)** | **(N=200)** |
| For each of the vaccines listed below, please select all statement(s) that generally apply to you. (n, %) | | | | | |
| Influenza | | | | | |
| I recommend this vaccine to my eligible adult patients | 653 (93.3) | 184 (92.5) | 142 (92.8) | 138 (93.2) | 189 (94.5) |
| My workplace stocks this vaccine for administration to my eligible adult patients | 442 (63.1) | 137 (68.8) | 68 (44.4) | 91 (61.5) | 146 (73.0) |
| None of these statements apply to me | 12 (1.7) | 1 (0.5) | 4 (2.6) | 4 (2.7) | 3 (1.5) |
| Pneumococcal disease | | | | | |
| I recommend this vaccine to my eligible adult patients | 645 (92.1) | 183 (92.0) | 146 (95.4) | 133 (89.9) | 183 (91.5) |
| My workplace stocks this vaccine for administration to my eligible adult patients | 398 (56.9) | 127 (63.8) | 51 (33.3) | 73 (49.3) | 147 (73.5) |
| None of these statements apply to me | 18 (2.6) | 2 (1.0) | 4 (2.6) | 7 (4.7) | 5 (2.5) |
| COVID-19 | | | | | |
| I recommend this vaccine to my eligible adult patients | 566 (80.9) | 161 (80.9) | 140 (91.5) | 113 (76.4) | 152 (76.0) |
| My workplace stocks this vaccine for administration to my eligible adult patients | 303 (43.3) | 76 (38.2) | 34 (22.2) | 47 (31.8) | 146 (73.0) |
| None of these statements apply to me | 66 (9.4) | 20 (10.1) | 9 (5.9) | 24 (16.2) | 13 (6.5) |
| RSV | | | | | |
| I recommend this vaccine to my eligible adult patients | 589 (84.1) | 171 (85.9) | 131 (85.6) | 121 (81.8) | 166 (83.0) |
| My workplace stocks this vaccine for administration to my eligible adult patients | 255 (36.4) | 59 (29.6) | 27 (17.6) | 28 (18.9) | 141 (70.5) |
| None of these statements apply to me | 65 (9.3) | 18 (9.0) | 17 (11.1) | 20 (13.5) | 10 (5.0) |
| Pertussis | | | | | |
| I recommend this vaccine to my eligible adult patients | 555 (79.3) | 176 (88.4) | 107 (69.9) | 122 (82.4) | 150 (75.0) |
| My workplace stocks this vaccine for administration to my eligible adult patients | 351 (50.1) | 108 (54.3) | 31 (20.3) | 72 (48.6) | 140 (70.0) |
| None of these statements apply to me | 80 (11.4) | 9 (4.5) | 35 (22.9) | 16 (10.8) | 20 (10.0) |
| Shingles | | | | | |
| I recommend this vaccine to my eligible adult patients | 618 (88.3) | 186 (93.5) | 119 (77.8) | 131 (88.5) | 182 (91.0) |
| My workplace stocks this vaccine for administration to my eligible adult patients | 293 (41.9) | 74 (37.2) | 27 (17.6) | 49 (33.1) | 143 (71.5) |
| None of these statements apply to me | 48 (6.9) | 4 (2.0) | 27 (17.6) | 12 (8.1) | 5 (2.5) |
| Hepatitis B | | | | | |
| I recommend this vaccine to my eligible adult patients | 521 (74.4) | 172 (86.4) | 98 (64.1) | 117 (79.1) | 134 (67.0) |
| My workplace stocks this vaccine for administration to my eligible adult patients | 333 (47.6) | 95 (47.7) | 30 (19.6) | 69 (46.6) | 139 (69.5) |
| None of these statements apply to me | 94 (13.4) | 7 (3.5) | 43 (28.1) | 21 (14.2) | 23 (11.5) |
| To what extent are your patients in the following age groups likely to get vaccinated with the vaccines that you recommend? (n, %) | | | | | |
| 18–49 years | | | | | |
| Very likely | 53 (7.6) | 19 (9.5) | 13 (8.5) | 14 (9.5) | 7 (3.5) |
| Somewhat likely | 321 (45.9) | 89 (44.7) | 59 (38.6) | 76 (51.4) | 97 (48.5) |
| Neither likely nor unlikely | 135 (19.3) | 42 (21.1) | 27 (17.6) | 25 (16.9) | 41 (20.5) |
| Somewhat unlikely | 106 (15.1) | 21 (10.6) | 29 (19.0) | 17 (11.5) | 39 (19.5) |
| Very unlikely | 61 (8.7) | 19 (9.5) | 18 (11.8) | 13 (8.8) | 11 (5.5) |
| I do not recommend vaccines to this age group | 24 (3.4) | 9 (4.5) | 7 (4.6) | 3 (2.0) | 5 (2.5) |
| 50–59 years | | | | | |
| Very likely | 125 (17.9) | 36 (18.1) | 29 (19.0) | 30 (20.3) | 30 (15.0) |
| Somewhat likely | 419 (59.9) | 123 (61.8) | 81 (52.9) | 91 (61.5) | 124 (62.0) |
| Neither likely nor unlikely | 70 (10.0) | 17 (8.5) | 17 (11.1) | 14 (9.5) | 22 (11.0) |
| Somewhat unlikely | 57 (8.1) | 12 (6.0) | 19 (12.4) | 7 (4.7) | 19 (9.5) |
| Very unlikely | 13 (1.9) | 5 (2.5) | 4 (2.6) | 3 (2.0) | 1 (0.5) |
| I do not recommend vaccines to this age group | 16 (2.3) | 6 (3.0) | 3 (2.0) | 3 (2.0) | 4 (2.0) |
| 60–74 years | | | | | |
| Very likely | 399 (57.0) | 119 (59.8) | 79 (51.6) | 77 (52.0) | 124 (62.0) |
| Somewhat likely | 248 (35.4) | 68 (34.2) | 62 (40.5) | 60 (40.5) | 58 (29.0) |
| Neither likely nor unlikely | 19 (2.7) | 6 (3.0) | 4 (2.6) | 5 (3.4) | 4 (2.0) |
| Somewhat unlikely | 15 (2.1) | 3 (1.5) | 3 (2.0) | 3 (2.0) | 6 (3.0) |
| Very unlikely | 14 (2.0) | 2 (1.0) | 4 (2.6) | 1 (0.7) | 7 (3.5) |
| I do not recommend vaccines to this age group | 5 (0.7) | 1 (0.5) | 1 (0.7) | 2 (1.4) | 1 (0.5) |
| ≥75 years | | | | | |
| Very likely | 484 (69.1) | 143 (71.9) | 104 (68.0) | 92 (62.2) | 145 (72.5) |
| Somewhat likely | 162 (23.1) | 48 (24.1) | 34 (22.2) | 43 (29.1) | 37 (18.5) |
| Neither likely nor unlikely | 18 (2.6) | 2 (1.0) | 3 (2.0) | 7 (4.7) | 6 (3.0) |
| Somewhat unlikely | 10 (1.4) | 2 (1.0) | 4 (2.6) | 2 (1.4) | 2 (1.0) |
| Very unlikely | 20 (2.9) | 2 (1.0) | 7 (4.6) | 2 (1.4) | 9 (4.5) |
| I do not recommend vaccines to this age group | 6 (0.9) | 2 (1.0) | 1 (0.7) | 2 (1.4) | 1 (0.5) |
| Generally, are your eligible adult patients receptive or resistant to your recommendations for each of the following vaccines? (n, %) | | | | | |
| Influenza | | | | | |
| Very receptive | 270 (38.6) | 73 (36.7) | 68 (44.4) | 39 (26.4) | 90 (45.0) |
| Somewhat receptive | 339 (48.4) | 103 (51.8) | 68 (44.4) | 86 (58.1) | 82 (41.0) |
| Neither receptive nor resistant | 39 (5.6) | 8 (4.0) | 7 (4.6) | 6 (4.1) | 18 (9.0) |
| Somewhat resistant | 42 (6.0) | 13 (6.5) | 9 (5.9) | 12 (8.1) | 8 (4.0) |
| Very resistant | 6 (0.9) | 2 (1.0) | 1 (0.7) | 2 (1.4) | 1 (0.5) |
| I do not recommend this vaccine | 4 (0.6) | 0 (0.0) | 0 (0.0) | 3 (2.0) | 1 (0.5) |
| Pneumococcal disease | | | | | |
| Very receptive | 291 (41.6) | 91 (45.7) | 76 (49.7) | 49 (33.1) | 75 (37.5) |
| Somewhat receptive | 336 (48.0) | 92 (46.2) | 68 (44.4) | 77 (52.0) | 99 (49.5) |
| Neither receptive nor resistant | 51 (7.3) | 9 (4.5) | 8 (5.2) | 13 (8.8) | 21 (10.5) |
| Somewhat resistant | 14 (2.0) | 6 (3.0) | 0 (0.0) | 4 (2.7) | 4 (2.0) |
| Very resistant | 3 (0.4) | 1 (0.5) | 1 (0.7) | 1 (0.7) | 0 (0.0) |
| I do not recommend this vaccine | 5 (0.7) | 0 (0.0) | 0 (0.0) | 4 (2.7) | 1 (0.5) |
| COVID-19 | | | | | |
| Very receptive | 80 (11.4) | 25 (12.6) | 27 (17.6) | 6 (4.1) | 22 (11.0) |
| Somewhat receptive | 256 (36.6) | 69 (34.7) | 64 (41.8) | 55 (37.2) | 68 (34.0) |
| Neither receptive nor resistant | 124 (17.7) | 37 (18.6) | 24 (15.7) | 18 (12.2) | 45 (22.5) |
| Somewhat resistant | 180 (25.7) | 46 (23.1) | 32 (20.9) | 51 (34.5) | 51 (25.5) |
| Very resistant | 47 (6.7) | 16 (8.0) | 6 (3.9) | 14 (9.5) | 11 (5.5) |
| I do not recommend this vaccine | 13 (1.9) | 6 (3.0) | 0 (0.0) | 4 (2.7) | 3 (1.5) |
| RSV | | | | | |
| Very receptive | 86 (12.3) | 19 (9.5) | 24 (15.7) | 14 (9.5) | 29 (14.5) |
| Somewhat receptive | 331 (47.3) | 93 (46.7) | 72 (47.1) | 59 (39.9) | 107 (53.5) |
| Neither receptive nor resistant | 188 (26.9) | 56 (28.1) | 44 (28.8) | 44 (29.7) | 44 (22.0) |
| Somewhat resistant | 76 (10.9) | 24 (12.1) | 8 (5.2) | 25 (16.9) | 19 (9.5) |
| Very resistant | 2 (0.3) | 1 (0.5) | 0 (0.0) | 1 (0.7) | 0 (0.0) |
| I do not recommend this vaccine | 17 (2.4) | 6 (3.0) | 5 (3.3) | 5 (3.4) | 1 (0.5) |
| Pertussis | | | | | |
| Very receptive | 132 (18.9) | 39 (19.6) | 38 (24.8) | 23 (15.5) | 32 (16.0) |
| Somewhat receptive | 310 (44.3) | 99 (49.7) | 57 (37.3) | 67 (45.3) | 87 (43.5) |
| Neither receptive nor resistant | 176 (25.1) | 51 (25.6) | 31 (20.3) | 37 (25.0) | 57 (28.5) |
| Somewhat resistant | 48 (6.9) | 6 (3.0) | 10 (6.5) | 15 (10.1) | 17 (8.5) |
| Very resistant | 3 (0.4) | 1 (0.5) | 0 (0.0) | 1 (0.7) | 1 (0.5) |
| I do not recommend this vaccine | 31 (4.4) | 3 (1.5) | 17 (11.1) | 5 (3.4) | 6 (3.0) |
| Shingles | | | | | |
| Very receptive | 222 (31.7) | 55 (27.6) | 44 (28.8) | 39 (26.4) | 84 (42.0) |
| Somewhat receptive | 349 (49.9) | 109 (54.8) | 75 (49.0) | 76 (51.4) | 89 (44.5) |
| Neither receptive nor resistant | 79 (11.3) | 27 (13.6) | 15 (9.8) | 14 (9.5) | 23 (11.5) |
| Somewhat resistant | 29 (4.1) | 6 (3.0) | 7 (4.6) | 13 (8.8) | 3 (1.5) |
| Very resistant | 3 (0.4) | 2 (1.0) | 0 (0.0) | 1 (0.7) | 0 (0.0) |
| I do not recommend this vaccine | 18 (2.6) | 0 (0.0) | 12 (7.8) | 5 (3.4) | 1 (0.5) |
| Hepatitis B | | | | | |
| Very receptive | 102 (14.6) | 27 (13.6) | 31 (20.3) | 18 (12.2) | 26 (13.0) |
| Somewhat receptive | 257 (36.7) | 91 (45.7) | 59 (38.6) | 58 (39.2) | 49 (24.5) |
| Neither receptive nor resistant | 229 (32.7) | 59 (29.6) | 34 (22.2) | 44 (29.7) | 92 (46.0) |
| Somewhat resistant | 62 (8.9) | 15 (7.5) | 7 (4.6) | 17 (11.5) | 23 (11.5) |
| Very resistant | 9 (1.3) | 4 (2.0) | 0 (0.0) | 2 (1.4) | 3 (1.5) |
| I do not recommend this vaccine | 41 (5.9) | 3 (1.5) | 22 (14.4) | 9 (6.1) | 7 (3.5) |
| For vaccines that are not stocked at your workplace, approximately what percentage of your adult patients do you think follow through with getting the vaccines that you recommend? (n, %) | | | | | |
|  | N=699 | N=198 | N=153 | N=148 | N=200 |
| 0% | 6 (0.9) | 1 (0.5) | 0 (0.0) | 1 (0.7) | 4 (2.0) |
| 1–25% | 148 (21.2) | 33 (16.7) | 19 (12.4) | 29 (19.6) | 67 (33.5) |
| 26–50% | 191 (27.3) | 63 (31.8) | 37 (24.2) | 51 (34.5) | 40 (20.0) |
| 51–75% | 182 (26.0) | 54 (27.3) | 60 (39.2) | 40 (27.0) | 28 (14.0) |
| 76–100% | 69 (9.9) | 29 (14.6) | 16 (10.5) | 12 (8.1) | 12 (6.0) |
| I do not recommend vaccines | 4 (0.6) | 0 (0.0) | 1 (0.7) | 2 (1.4) | 1 (0.5) |
| Not applicable (my workplace stocks all adult vaccines | 58 (8.3) | 12 (6.1) | 6 (3.9) | 10 (6.8) | 30 (15.0) |
| Don’t know | 41 (5.9) | 6 (3.0) | 14 (9.2) | 3 (2.0) | 18 (9.0) |
| Are you (or someone at your workplace) able to recommend and/or administer adult vaccines that are FDA-approved but that do not have a recommendation from the CDC’s ACIP? (n, %) | | | | | |
|  | N=699 | N=198 | N=153 | N=148 | N=200 |
| Yes, and I sometimes do this. | 233 (33.3) | 75 (37.9) | 60 (39.2) | 49 (33.1) | 49 (24.5) |
| Yes, but I don't do this because I prefer to follow ACIP recommendations. | 213 (30.5) | 84 (42.4) | 50 (32.7) | 37 (25.0) | 42 (21.0) |
| No, my workplace requires an ACIP recommendation to be able to vaccinate. | 238 (34.0) | 39 (19.7) | 35 (22.9) | 58 (39.2) | 106 (53.0) |
| I do not recommend any vaccinations to adult patients. | 15 (2.1) | 0 (0.0) | 8 (5.2) | 4 (2.7) | 3 (1.5) |

Abbreviations: ACIP, Advisory Committee on Immunization Practices; CDC, Centers for Disease Control and Prevention; COVID-19, coronavirus disease 2019; HCP, healthcare professional; NP, nurse practitioner; PA, physician assistant; PCP, primary care physician; RSV, respiratory syncytial virus.
