# Supplementary material for: US healthcare professionals’ knowledge, attitudes, and practices regarding RSV disease and vaccination in adults during the 2024–2025 RSV season
Source: PLoS One. 2026 Jul 22;21(7):e0353266. doi: 10.1371/journal.pone.0353266 (PMC13390937; doi:10.1371/journal.pone.0353266)
Supplement: S1 File — (DOCX) [file pone.0353266.s008.docx]

**HCP Survey Questionnaire**

**Screening questions**

S0. Do we have your permission to collect your answers to the eligibility questions to see whether you qualify to participate in the study?

☐ Yes

☐ No 🡪Not eligible

S1. Please indicate your primary medical profession.

☐ Primary care physician (e.g., family medicine, internal medicine)

☐ Specialist physician: Cardiologist

☐ Specialist physician: Endocrinologist

☐ Specialist physician: Pulmonologist

☐ Nurse practitioner

☐ Physician assistant

☐ Registered pharmacist

☐ None of these 🡪Not eligible

S2. As <S1 response: “a primary care physician”, “a cardiologist”, “an endocrinologist”, “a pulmonologist”, “a nurse practitioner”, “a physician assistant”, or “a pharmacist”>, is your current status any of the following: a resident, fellow, or trainee?

☐ Yes 🡪Not eligible

☐ No

If S1 = pharmacist

S3. As a pharmacist, are you certified with the proper legal authority to administer immunizations?

☐ Yes

☐ No 🡪Not eligible

S4. In which state do you currently work? If your work location is in more than one state, please select the state where you consider your primary workplace to be located. ____________ [Drop-down menu that includes all 50 states and Washington, DC; not eligible if Maine, Massachusetts, Minnesota, or Vermont is selected]

S5. In an average week, how many hours do you work in [if S1= “Registered pharmacist”, “a pharmacy”, else if S1 ≠ “Registered pharmacist”, “direct patient care”]?

☐ Less than 10 hours 🡪Not eligible

☐ 10 to 20 hours 🡪Not eligible

☐ 21 to 30 hours

☐ 31 to 40 hours

☐ More than 40 hours

S6. Overall, how many years have you been working as <S1 response: “a primary care physician”, “a cardiologist”, “an endocrinologist”, “a pulmonologist”, “a nurse practitioner”, “a physician assistant”, or “a pharmacist”>?

☐ Less than 2 years 🡪Not eligible

☐ 2 to 10 years

☐ 11 to 20 years

☐ 21 to 30 years

☐ More than 30 years

If S1 ≠ “Registered pharmacist”

S7. What is your primary work environment?

☐ Private practice, solo

☐ Private practice, single specialty group

☐ Private practice, multispecialty group

☐ Managed care or health maintenance organization (HMO) practice

☐ Outpatient clinic (e.g., ambulatory care center, urgent care center)

☐ Academic medical center

☐ Hospital inpatient service 🡪Not eligible

☐ Other (e.g., research, administration) 🡪Not eligible

If S1 = “Registered pharmacist”

S8. What is your primary work environment?

☐ Large pharmacy chain (e.g., CVS, Walgreens, Rite Aid)

☐ Grocery store/chain (e.g., Albertson’s, Publix, HEB, Kroger, Wegmans)

☐ Mass merchant (e.g., Walmart, Costco)

☐ Regional pharmacy chain (e.g., Kinney Drugs, Thrifty White)

☐ Independent pharmacy

☐ Mail-order pharmacy 🡪Not eligible

☐ Hospital-based inpatient, outpatient, or clinic pharmacy 🡪Not eligible

☐ Specialty pharmacy (e.g., compounding, cannabis, nuclear) 🡪Not eligible

☐ Other 🡪Not eligible

If S1 ≠ “Registered pharmacist”

S9. Do you spend most of your time working in an emergency department or a surgical setting?

☐ Yes 🡪Not eligible

☐ No

If S1 ≠ “Registered pharmacist”

S10. In the past week, have you provided care for at least 1 adult patient aged 50 years or older?

☐ Yes

☐ No 🡪Not eligible

If S1 = “Registered pharmacist”

S11. In the past week, have you personally interacted with at least 1 adult patient aged 50 years or older?

☐ Yes

☐ No 🡪Not eligible

**Screening questions**

| **This set of general questions is about your primary workplace and patients.** |
| --- |

1. Which category best describes the location of your primary workplace?

☐ Urban (i.e., city or metropolitan area)

☐ Suburban (i.e., large residential area that surrounds a main city)

☐ Rural

1. Please estimate the percentage of adult patients (aged 18 years and older) seen in your workplace that fall under each race category listed. *Your best estimates are fine*.

(Enter whole numbers for each row. Your answers must add up to 100% before you can proceed.)

| Race | % of adult patients |
| --- | --- |
| White | % |
| Black or African American | % |
| Asian | % |
| Another race not listed | % |
| **Total** | <autosum>% |

1. What percentage of adult patients (aged 18 years and older) seen in your workplace are Hispanic, Latina(o), Latine, or Latinx? *Your best estimate is fine*.

*__________* % of adult patients

1. Please indicate the approximate percentage of adult patients (aged 18 years and older) seen in your workplace who have each of the following types of health insurance. *Your best estimates are fine*.

(Enter whole numbers for each row. Your answers must add up to 100% before you can proceed.)

| Health Insurance Type | % of adult patients |
| --- | --- |
| Commercial/private insurance | % |
| Medicaid | % |
| Medicare | % |
| Other health insurance | % |
| No health insurance | % |
| **Total** | <autosum>% |

1. Please enter the approximate percentage of the patients you personally <if S1 ≠ “Registered pharmacist”, “evaluate and/or treat”; if S1 = “Registered pharmacist”, “interact with”> in each of the following age groups. Please consider only yourself and no other healthcare professionals at your workplace. You may include telehealth visits as part of this estimate.

(Enter whole numbers for each row.)

| Age Group | % of patients |
| --- | --- |
| Younger than 18 years | % |
| 18-59 years | % |
| 60 years and older | % |
| **Total** | <autosum>% |

1. During an average week, about how many adult patients aged 18 years and older do you personally <if S1 ≠ “Registered pharmacist”, “evaluate and/or treat”; if S1 = “Registered pharmacist”, “interact with”>? Please consider only yourself and no other healthcare professionals at your workplace. You may include telehealth visits as part of this estimate.

(Enter whole number.)

__________ # of adult patients aged 18 years and older

| **This set of questions is about your experience with respiratory infections.** |
| --- |

1. During an average week within the respiratory infection season, approximately how many adult patients aged 18 years and older do you see with respiratory infections?

☐ None

☐ Less than 10

☐ 10-25

☐ 26-50

☐ More than 50

1. Which of the following best describes your familiarity with respiratory syncytial virus (RSV) disease?

☐ Never heard of RSV disease 🡪Skip to Q17

☐ Heard of RSV disease but do not know much about it

☐ Know some information about RSV disease

☐ Very familiar with RSV disease <Display if S1 ≠ “Registered pharmacist”: “but **have not managed or diagnosed RSV disease**”>

<Display if S1 ≠ “Registered pharmacist”> ☐ Very familiar with RSV disease and **have managed or diagnosed RSV disease**

If S1 ≠ “Registered pharmacist”

1. How often do you test (or order a lab test) for RSV in your adult patients (aged 18 years and older) with respiratory infections?

☐ Always

☐ Often

☐ Sometimes

☐ Rarely

☐ Never

Hide if Q7 = “None”

You previously indicated that you see around <Q7 response> adult patients with respiratory infections in an average week during the respiratory infection season.

1. Approximately what percentage of these adult patients with respiratory infections do you suspect have RSV?

☐ None

☐ Less than 5%

☐ 5-14%

☐ 15-24%

☐ 25-49%

☐ 50% or more

☐ Don’t know

1. For the age groups below, to what extent do you feel that age alone is a risk factor for severe RSV disease?

| Age group | No risk | Low risk | Moderate risk | High risk | Don’t know |
| --- | --- | --- | --- | --- | --- |
| 18-49 years | ☐ | ☐ | ☐ | ☐ | ☐ |
| 50-59 years | ☐ | ☐ | ☐ | ☐ | ☐ |
| 60-74 years | ☐ | ☐ | ☐ | ☐ | ☐ |
| 75+ years | ☐ | ☐ | ☐ | ☐ | ☐ |

1. For the age groups below, what percentage of adult patients do you think are at increased risk for severe RSV disease (e.g., due to age and/or presence of certain medical conditions that may place them at increased risk)?

(Enter whole numbers for each row.)

| Age group | % of patients in each age group who are at increased risk of severe RSV disease |
| --- | --- |
| 18-49 years | % |
| 50-59 years | % |
| 60-74 years | % |
| 75+ years | % |

1. In patients aged 60 years and older with RSV, approximately what percentage of medically attended cases (i.e., cases seen by a doctor or other healthcare professional) do you think result in hospitalization?

☐ <5%

☐ 5-14%*

☐ 15-29%

☐ 30% or more

☐ Don’t know

1. In patients aged 60 years and older with RSV, approximately what percentage of hospitalized cases do you think experience in-hospital death?

☐ <2%

☐ 4-8%*

☐ 15-20%

☐ 25% or more

☐ Don’t know

1. For the health conditions/patient characteristics below, to what extent is each item a risk factor for severe RSV disease among adult patients?

| Health conditions/patient characteristics | No risk | Low risk | Moderate risk | High risk | Don’t know |
| --- | --- | --- | --- | --- | --- |
| Chronic lung or respiratory disease *(e.g., chronic obstructive pulmonary disease, emphysema, asthma, interstitial lung disease, or cystic fibrosis)* | ☐ | ☐ | ☐ | ☐ | ☐ |
| Severe obesity *(body mass index ≥40kg/m^2^)* | ☐ | ☐ | ☐ | ☐ | ☐ |
| Chronic cardiovascular disease *(e.g., heart failure, coronary artery disease, or congenital heart disease [excluding isolated hypertension])* | ☐ | ☐ | ☐ | ☐ | ☐ |
| Moderate or severe immune compromise | ☐ | ☐ | ☐ | ☐ | ☐ |
| Diabetes mellitus complicated *by chronic kidney disease, neuropathy, retinopathy, or other end-organ damage, or requiring treatment with insulin or sodium-glucose cotransporter-2 (SGLT2) inhibitor* | ☐ | ☐ | ☐ | ☐ | ☐ |
| Neurological or neuromuscular conditions causing impaired airway clearance or respiratory muscle weakness *(e.g., poststroke dysphagia, amyotrophic lateral sclerosis, or muscular dystrophy)* | ☐ | ☐ | ☐ | ☐ | ☐ |
| End-stage renal disease *or dependence on hemodialysis or other renal replacement therapy* | ☐ | ☐ | ☐ | ☐ | ☐ |
| Chronic liver disease *(e.g., cirrhosis)* | ☐ | ☐ | ☐ | ☐ | ☐ |
| Residence in a nursing home | ☐ | ☐ | ☐ | ☐ | ☐ |
| Frailty | ☐ | ☐ | ☐ | ☐ | ☐ |
| Chronic hematologic conditions *(e.g., sickle cell disease or thalassemia)* | ☐ | ☐ | ☐ | ☐ | ☐ |

1. To what extent do you agree with the following statement regarding RSV disease?

|  | Strongly agree | Agree | Disagree | Strongly disagree | Don’t know |
| --- | --- | --- | --- | --- | --- |
| RSV disease impacts patients’ underlying conditions (e.g., COPD, asthma). | ☐ | ☐ | ☐ | ☐ | ☐ |

| **This next set of questions are about your perspectives on vaccines.** |
| --- |

1. To what extent are your patients in the following age groups likely to get vaccinated with the vaccines that you recommend?

| Patient age group | Very likely | Somewhat likely | Neither likely nor unlikely | Somewhat unlikely | Very unlikely | I do not recommend vaccines to this age group |
| --- | --- | --- | --- | --- | --- | --- |
| 18-49 years | ☐ | ☐ | ☐ | ☐ | ☐ | ☐ |
| 50-59 years | ☐ | ☐ | ☐ | ☐ | ☐ | ☐ |
| 60-74 years | ☐ | ☐ | ☐ | ☐ | ☐ | ☐ |
| 75+ years | ☐ | ☐ | ☐ | ☐ | ☐ | ☐ |

1. Generally, are your eligible adult patients receptive or resistant to your recommendations for each of the following vaccines?

|  | Very receptive | Somewhat receptive | Neither receptive nor resistant | Somewhat resistant | Very resistant | I do not recommend this vaccine |
| --- | --- | --- | --- | --- | --- | --- |
| Influenza | ☐ | ☐ | ☐ | ☐ | ☐ | ☐ |
| Pneumococcal disease | ☐ | ☐ | ☐ | ☐ | ☐ | ☐ |
| COVID-19 | ☐ | ☐ | ☐ | ☐ | ☐ | ☐ |
| RSV | ☐ | ☐ | ☐ | ☐ | ☐ | ☐ |
| Pertussis | ☐ | ☐ | ☐ | ☐ | ☐ | ☐ |
| Shingles | ☐ | ☐ | ☐ | ☐ | ☐ | ☐ |
| Hepatitis B | ☐ | ☐ | ☐ | ☐ | ☐ | ☐ |

1. Are you (or someone at your workplace) able to recommend and/or administer adult vaccines that are FDA-approved but that do not have a recommendation from the Centers for Disease Control and Prevention’s (CDC) Advisory Committee on Immunization Practices (ACIP)?

☐ Yes, and I sometimes do this.

☐ Yes, but I don’t do this because I prefer to follow ACIP recommendations.

☐ No, my workplace requires an ACIP recommendation to be able to vaccinate.

☐ I do not recommend any vaccinations to adult patients.

1. For each of the vaccines listed below, please select all statement(s) that generally apply to you.

|  | I *recommend* this vaccine to my eligible adult patients | My workplace stocks this vaccine for *administration* to my eligible adult patients | None of these statements apply to me |
| --- | --- | --- | --- |
| Influenza | ☐ | ☐ | ☐ |
| Pneumococcal disease | ☐ | ☐ | ☐ |
| COVID-19 | ☐ | ☐ | ☐ |
| RSV | ☐ | ☐ | ☐ |
| Pertussis | ☐ | ☐ | ☐ |
| Shingles | ☐ | ☐ | ☐ |
| Hepatitis B | ☐ | ☐ | ☐ |

1. For vaccines that are not stocked at your workplace, approximately what percentage of your adult patients do you think follow through with getting the vaccines that you recommend?

☐ 0%

☐ 1-25%

☐ 26-50%

☐ 51-75%

☐ 76-100%

☐ I do not recommend vaccines

☐ Not applicable (my workplace stocks all adult vaccines)

☐ Don’t know

If Q8 = “Never heard of RSV disease”, skip to Q37

1. To your knowledge, how many RSV vaccines are currently FDA-approved for use in adults?

☐ 0 🡪 Skip to Q25

☐ 1

☐ 2

☐ 3*

☐ 4

☐ 5 or more

☐ Don’t know

1. From what you know, for which of the following non-pregnant adult groups has the FDA approved RSV vaccine(s)? Consider increased risk populations to be those who are at increased risk for lower respiratory tract disease caused by RSV.

| Age group | Not FDA-approved | FDA-approved only for adults in this age range who are at increased risk | FDA-approved for all adults in this age range | Don’t know |
| --- | --- | --- | --- | --- |
| 18-49 years | ☐ | ☐* | ☐ | ☐ |
| 50-59 years | ☐ | ☐* | ☐ | ☐ |
| 60-74 years | ☐ | ☐ | ☐* | ☐ |
| 75+ years | ☐ | ☐ | ☐* | ☐ |

1. From what you know, for which of the following non-pregnant adult groups has the Centers for Disease Control and Prevention’s (CDC’s) Advisory Committee on Immunization Practices (ACIP) recommended RSV vaccines? Consider increased risk populations to be those who are at increased risk for lower respiratory tract disease caused by RSV.

| Age group | Not ACIP-recommended | ACIP-recommended only for adults in this age range who are at increased risk | ACIP-recommended for all adults in this age range | Don’t know |
| --- | --- | --- | --- | --- |
| 18-49 years | ☐* | ☐ | ☐ | ☐ |
| 50-59 years | ☐* | ☐ | ☐ | ☐ |
| 60-74 years | ☐ | ☐* | ☐ | ☐ |
| 75+ years | ☐ | ☐ | ☐* | ☐ |

1. For the age groups below, how beneficial do you think it is to have an RSV vaccination available and included in the CDC’s ACIP recommendations?

|  | Not at all beneficial | Somewhat beneficial | Beneficial | Very beneficial | Don’t know |
| --- | --- | --- | --- | --- | --- |
| All adults aged 18-49 years | ☐ | ☐ | ☐ | ☐ | ☐ |
| Adults aged 18-49 years who are at increased risk | ☐ | ☐ | ☐ | ☐ | ☐ |
| All adults aged 50-59 years | ☐ | ☐ | ☐ | ☐ | ☐ |
| Adults aged 50-59 years who are at increased risk | ☐ | ☐ | ☐ | ☐ | ☐ |
| All adults aged 60-74 years | ☐ | ☐ | ☐ | ☐ | ☐ |
| Adults aged 60-74 years who are at increased risk | ☐ | ☐ | ☐ | ☐ | ☐ |

1. To your knowledge, what percentage of RSV lower respiratory tract disease cases would the currently available RSV vaccines prevent in adults aged 60 years and older during the first season after vaccination (i.e., what is the vaccine efficacy)?

☐ <35%

☐ 35-54%

☐ 55-74%

☐ 75% or more*

☐ Don’t know

1. Which of the following approaches or processes do you currently use to inform your decision-making regarding recommending **RSV vaccination** in adult patients aged 60 years and older? *Please select all that apply.*

☐ Clinical judgment

☐ Shared clinical decision-making

☐ Clinical decision support systems (e.g., EMR reminder, immunization alerts)

☐ Treatment protocols at my workplace

☐ My patients ask about RSV vaccination

☐ A state IIS that includes a reminder/recall for vaccinations

☐ My patients complete a questionnaire in the waiting room or prior to the visit that includes the patient’s history of vaccinations

☐ Training to keep updated on immunization recommendations and/or guidelines

☐ Annual immunization schedules included on the CDC website

☐ ACIP recommendations

☐ Other professional guidelines (e.g., AACE, AAFP, AANP, AAPA, ACC, ACP, APhA, CHEST)

☐ None of the above [Exclusive]

1. When deciding on recommending an RSV vaccination to adult patients, to what extent do you follow the CDC’s Advisory Committee on Immunization Practices (ACIP) recommendations?

☐ Always

☐ Often

☐ Sometimes

☐ Rarely

☐ Never

☐ I am not familiar with the ACIP recommendations for RSV vaccination

1. Which of the following are barriers or concerns related to recommending or administering RSV vaccination to an adult patient aged 60 years or older?

☐ Contraindications (e.g., history of allergic reactions)

☐ I have concerns about the effectiveness of the vaccine

☐ Patients’ concerns about the effectiveness of the vaccine

☐ I have concerns about the safety of the vaccine

☐ Patients’ concerns about the safety of the vaccine

☐ Patients’ medical insurance does not cover the vaccine

☐ Patients’ lack of awareness of RSV

☐ I do not believe RSV is as severe as other conditions and would prioritize other vaccines instead

☐ My workplace does not focus on recommending or administering vaccinations

☐ Patients refuse to be vaccinated or are hesitant

☐ Patients don’t think RSV is a risk to them

☐ I do not regularly review patients’ vaccination records as part of their visit

☐ None of the above [Exclusive]

1. Since September 1, 2024, please estimate the percentage of adult patients in each group listed below with whom you discussed, recommended, and/or administered RSV vaccination. Please consider only your personal interactions and not the experiences of other healthcare professionals in your workplace. Consider increased risk populations to be those who are at increased risk for lower respiratory tract disease caused by RSV. Your best estimate is fine.

(Enter whole numbers for each row.)

| Patient age | Discussed RSV vaccination | Recommended RSV vaccination | Personally (or someone else at my workplace) administered RSV vaccination |
| --- | --- | --- | --- |
| Adults aged 50-59 years who are at increased risk | % | % | % |
| Healthy adults aged 60-74 years | % | % | % |
| Adults aged 60-74 years who are at increased risk | % | % | % |
| All adults aged 75 years and older | % | % | % |

1. What topics do you think are important to cover when discussing RSV vaccination with patients aged 60 years and older? *Please select all that apply*.

☐ Risk of getting RSV

☐ Risk for serious illness or complications from RSV

☐ Risk of death from RSV

☐ Risk of adverse side effects from vaccine

☐ Risk of transmitting RSV to others

☐ ACIP recommendations for the RSV vaccine

☐ Safety of the RSV vaccine

☐ Efficacy of the RSV vaccine

☐ Cost of the RSV vaccine

☐ My personal recommendation for the patient

☐ None of the above [Exclusive]

1. Reflecting on your experiences discussing RSV vaccination with patients, how often do patients in each of the following groups initiate the discussion?

| Age group | Most of  the time | Often | Sometimes | Rarely | I have not discussed RSV vaccination with this age group |
| --- | --- | --- | --- | --- | --- |
| 50-59 years | ☐ | ☐ | ☐ | ☐ | ☐ |
| 60-74 years | ☐ | ☐ | ☐ | ☐ | ☐ |
| 75+ years | ☐ | ☐ | ☐ | ☐ | ☐ |

1. To what extent do you agree with the following statements regarding RSV vaccinations?

|  | Strongly agree | Agree | Disagree | Strongly disagree | Don’t know |
| --- | --- | --- | --- | --- | --- |
| **[1]** Adults aged 50-59 years who are at increased risk of severe RSV disease should be included in ACIP recommendations for RSV vaccination. | ☐ | ☐ | ☐ | ☐ | ☐ |
| **[2]** The benefits of RSV vaccination outweigh the potential risks for adults aged 50-59 years who are at increased risk of severe RSV disease. | ☐ | ☐ | ☐ | ☐ | ☐ |
| **[3]** Adults aged 50-59 years who are at increased risk of severe RSV disease can get the RSV vaccine even though the ACIP has not provided a recommendation in this group. | ☐ | ☐ | ☐ | ☐ | ☐ |
| **[4]** I am concerned that my adult patients aged 50-59 years who are at increased risk of severe RSV disease won’t be able to get the RSV vaccine because the ACIP has not provided a recommendation in this group. | ☐ | ☐ | ☐ | ☐ | ☐ |

1. Does your workplace stock RSV vaccine(s) to administer to patients?

☐ Yes, year round

☐ Yes, seasonally

☐ No

1. To what extent do you agree that stocking RSV vaccine(s) for administration at your workplace <if Q34 = “Yes, year round” or “Yes, seasonally”, display “helps”; if Q34 = “No”, display “would help”> to ensure at-risk individuals get vaccinated?

☐ Strongly agree

☐ Agree

☐ Neither agree nor disagree

☐ Disagree

☐ Strongly disagree

1. Among patients to whom you’ve recommended RSV vaccination, what percentage in each of the following age groups do you think have followed through and gotten vaccinated?

(Enter whole numbers for each row or select one of the other response options if you don’t know or if you do not recommend RSV vaccination to this age group.)

| Age group | % of patients that follow through with my RSV vaccination recommendation | Don’t know | I do not recommend RSV vaccination to this age group |
| --- | --- | --- | --- |
| 50-59 years | % | ☐ | ☐ |
| 60-74 years | % | ☐ | ☐ |
| 75+ years | % | ☐ | ☐ |

| **These final questions are about you.** |
| --- |

1. What is your age?

_________

1. Which of the following best describes your race and ethnicity? *Please select all that apply*.

☐ White

☐ Hispanic or Latino

☐ Black or African American

☐ Asian

☐ American Indian or Alaska Native

☐ Middle Eastern or North African

☐ Native Hawaiian or Pacific Islander

☐ Other race or ethnicity

1. What is your gender identity?

☐ Male

☐ Female

☐ A gender identity not listed
